# Identification of Molecular Pathways Underlying Mn(II) Oxidation in *Pseudomonas resinovorans*

**DOI:** 10.1111/1462-2920.70423

**Published:** 2026-10-01

**Authors:** Ainelén Piazza, Lucia Parra, Marilina Gaffuri, Jacob G. Malone, Jorgelina Ottado, Natalia Gottig

**Affiliations:** ^1^ Department of Molecular Microbiology John Innes Centre Norwich UK; ^2^ The Centre for Microbial Interactions Norwich UK; ^3^ Instituto de Biología Molecular y Celular de Rosario Consejo Nacional de Investigaciones Científicas y Técnicas, Universidad Nacional de Rosario Rosario Argentina; ^4^ School of Biological Sciences University of East Anglia Norwich UK

**Keywords:** biofilm, biological filtration, c‐di‐GMP, manganese, oxidation, *Pseudomonas resinovorans*, two‐component systems

## Abstract

Manganese oxidation in *Pseudomonas resinovorans* strain MOB‐513 has been previously linked to biofilm formation via the second messenger bis‐(3′–5′)‐cyclic dimeric GMP (c‐di‐GMP), although the specific molecular components mediating this regulation have not yet been identified. Here we investigate the genetic network underlying Mn(II) oxidation in MOB‐513. After transposon mutagenesis, several mutants with differences in Mn(II) oxidation were identified. Targeted analysis of the non‐oxidising mutants with Tn insertion disrupting *pilC*, *algR*, and *hk05390* genes, encoding a core Type IV pilus (Tfp) component, a regulator of pilus biogenesis, and an uncharacterised histidine kinase, respectively, revealed that their phenotype correlated with impaired biofilm formation, reduced intracellular c‐di‐GMP levels, and decreased Tfp production. We identified two regulators associated with a c‐di‐GMP‐dependent pathway linked to Mn(II) oxidation in MOB‐513. The hyper‐oxidising strain containing a transposon insertion in the promoter region of the *dgc05792* gene showed upregulation of multiple Mn(II)‐oxidising enzymes. RR05389 further modulates intracellular c‐di‐GMP levels and Mn(II) oxidation and functions as a putative cognate response regulator with the histidine kinase HK05390. Together, these results expand our understanding of the molecular pathways underlying Mn(II) oxidation and identify new targets for optimising biological manganese removal.

## Graphical abstract

Transposon mutagenesis in biofilter‐associated *Pseudomonas resinovorans* MOB‐513 identifies a c‐di‐GMP‐dependent regulatory network involving type IV pili, two‐component systems, and diguanylate cyclases that controls Mn(II) oxidation and biofilm formation, providing new insight into microbial manganese cycling and biofilter performance.

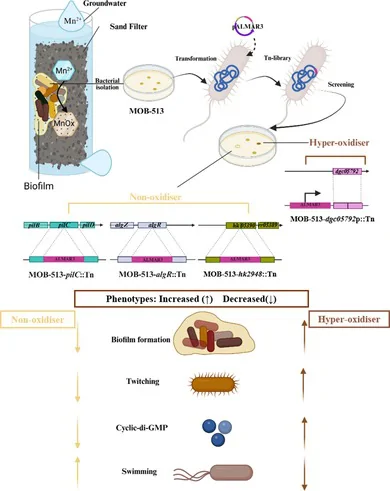

## 1 Introduction

Manganese is a highly abundant metal in the Earth's crust that frequently leaches into groundwater, an important source of drinking water, where it is present primarily as dissolved Mn(II). This metal impairs water quality by causing aesthetic, organoleptic, and operational problems, interfering with disinfection processes, and promoting corrosion and clogging of distribution systems (Sly et al. 1990). In addition, Mn(II) exposure has been associated with an increased risk of adverse health effects (Sly et al. 1990; Whelton et al. 2007; Avila et al. 2013). Consequently, effective removal of Mn(II) from drinking water is a critical public health concern.

Biological sand filtration is widely used as an efficient, low‐cost, and eco‐friendly technology for removing manganese from groundwater (Pacini et al. 2005). In these systems, water undergoes aeration followed by gravel and sand filtration stages. Efficient Mn(II) removal depends on manganese‐oxidising bacteria (MOB) (Piazza et al. 2019), that convert soluble Mn(II) into insoluble Mn(III/IV) oxides (MnOx) retained within the filter matrix.

MOB are typically present in groundwater and colonise sand filters by attaching to the matrix and forming biofilms. This biofilm‐forming capability is crucial for filter maturation, as it enables bacteria retention, sustained Mn(II) oxidation, and accumulation of MnOx on the sand surface (Piazza et al. 2019; Ciancio Casalini et al. 2022).

In practice, slow or incomplete Mn(II) removal has been associated with the absence or low abundance of MOB in groundwater (Pacini et al. 2005). To address this limitation, we previously isolated and characterised several MOB, mainly from the genus *Pseudomonas*, selecting strains with strong adhesion, robust biofilm formation, and high Mn(II)‐oxidising capacity (Piazza et al. 2019).

Bacterial Mn(II) oxidation is a widespread process in diverse environments and is mediated primarily by multicopper oxidases (MCOs), which oxidise Mn(II) using molecular oxygen, and manganese peroxidases (MOPs), which catalyse the reaction using hydrogen peroxide (Geszvain et al. 2012). However, the environmental factors that induce Mn oxidase expression and the potential advantages of Mn(II) oxidation for microbial life remain poorly understood.

One of the most extensively studied Mn(II)‐oxidising strains is *Pseudomonas putida* GB‐1, a bacterium that possesses two MCOs (MnxG and McoA), each capable of catalysing Mn(II) oxidation independently, as well as a manganese peroxidase (MopA) (Geszvain et al. 2016). The presence of multiple Mn oxidases within one organism adds complexity to deciphering the regulatory mechanisms underlying Mn(II) oxidation. Despite this challenge, several random transposon mutagenesis screens have identified specific gene groups required for Mn(II) oxidation or oxidase export in strain GB‐1, including the *ccm* operon for c‐type cytochrome synthesis (de Vrind et al. 1998; Caspi et al. 1998), which possibly mediates electron transfer from Mn(II) oxidation to the respiratory chain.

Therefore, the regulation of bacterial Mn(II) oxidation remains a fundamental question in environmental microbiology, with direct implications for the optimisation of bioremediation strategies. In GB‐1, a two‐component system (TCS) is known to be required for Mn(II) oxidation (Geszvain and Tebo 2010). TCS typically consists of a sensor histidine kinase (HK) and a response regulator (RR). In response to an environmental signal, the HK autophosphorylates at a conserved histidine residue within its kinase domain. The phosphoryl group is subsequently transferred to a RR, which often functions as a transcription factor. Phosphorylation of the RR modulates the transcription of downstream genes, thereby eliciting a physiological response to the environmental cue (Stock et al. 2000). In this strain, two histidine kinases MnxS1 and MnxS2 and a response regulator MnxR are essential for Mn(II) oxidation (Geszvain and Tebo 2010).

More recently, we identified a novel signalling pathway involved in Mn(II) oxidation in the environmental isolate *P. resinovorans* MOB‐513 (hereafter, MOB‐513) (Piazza et al. 2022). This pathway is regulated by the second messenger c‐di‐GMP, a molecule known to control the bacterial shift between motile and sessile lifestyles (Liu et al. 2024). Our findings revealed a direct link between c‐di‐GMP signalling, biofilm formation, and Mn(II) oxidation (Piazza et al. 2022).

Bacterial intracellular c‐di‐GMP levels are primarily controlled by two classes of enzymes: diguanylate cyclase (DGC), which synthesise c‐di‐GMP and contain GGDEF domains, and phosphodiesterase (PDE), which degrade c‐di‐GMP and possess EAL or HD‐GYP domains (Hengge et al. 2016; Jenal and Malone 2006). c‐di‐GMP exerts its regulatory effects by binding to diverse intracellular receptors, modulating cellular processes at transcriptional, translational, and post‐translational levels (Romling and Galperin 2015; Schirmer and Jenal 2009).

In MOB‐513 increased intracellular c‐di‐GMP levels, achieved through the ectopic expression of *dgcB*, a well‐characterised DGC gene from *Bordetella bronchiseptica* (Sisti et al. 2013), accelerate and enhance Mn(II) oxidation (Piazza et al. 2022). Under these conditions, c‐di‐GMP upregulates two manganese peroxidases, Mop04787 and Mop04788, identified as key enzymatic drivers of Mn(II) oxidation in MOB‐513 and modulates the activity of a c‐di‐GMP‐binding PilZ‐domain protein (PRES513_01015), which functions as a negative regulator. While these findings highlight a strong association between c‐di‐GMP in regulating Mn(II) oxidation, the broader impact of native DGCs and PDEs on c‐di‐GMP levels and additional pathways involved in Mn(II) oxidation remain largely unexplored.

In this study, we investigated the genetic determinants involved in Mn(II) oxidation in MOB‐513. To this end, we performed a random transposon mutagenesis screen and characterised mutants with abolished or enhanced Mn(II)‐oxidising capacity. This approach not only reinforced the essential link between c‐di‐GMP signalling, biofilm formation and Mn(II) oxidation, but also uncovered new key factors involved in this process. These included components of the Tfp, required for cell–cell interactions and biofilm development, as well as a previously uncharacterised TCS, and regulators likely involved in modulating the intracellular c‐di‐GMP levels associated with Mn(II) oxidation.

## 2 Methods

### 2.1 Strains, Plasmids, and Growth Conditions

Table S1 compiles the bacterial strains and plasmids used in this work. *P. resinovorans* MOB‐513 whole‐genome was re‐sequenced by Plasmidsaurus using the Oxford Nanopore long‐read technology, as previously described (Piazza et al. 2026), and the assembly is available in the DDBJ/EMBL/GenBank databases under accession number GCA_986265995. Given that the updated genome annotation differs from that used in our previous publication, we have included a (Table S2) that cross‐references the previous gene names with the updated annotations.

Cultures of *Escherichia coli* and MOB‐513 strains were grown in Lysogeny Broth (LB) medium at 37°C and 28°C, respectively (Bertani 1951). Where indicated, MOB‐513 was cultured in Lept medium with supplementation of 100 μM MnCl_2_, hereafter Lept+Mn (Boogerd and de Vrind 1987). Macrocolony biofilms of MOB‐513 strains were grown as previously described (Piazza et al. 2022); briefly, overnight LB cultures were diluted in LB to OD_600_ = 1.0 and 5 μL droplets were spotted onto Lept+Mn agar plates and incubated at 28°C over a 7‐day period.

*Pseudomonas* strains were transformed by electroporation. Briefly, approximately 200 ng of each plasmid DNA was mixed with 100 μL of electrocompetent cells, the mixture was pulsed at 2.5 kV, 25 μF and 200 Ω, and cells were recovered in 1 mL LB medium for 2 h at 28°C (Choi et al. 2006). *E. coli* strains were transformed by the standard CaCl_2_ chemical competence method previously described (Sambrook and Russell 2001). Antibiotics were supplemented, where appropriate, at the following final concentrations: Kanamycin (Km) at 25 μg/mL; Chloramphenicol (Cm) at 30 μg/mL; Gentamicin (Gm) at 20 μg/mL; Streptomycin (Sp), 25 μg/mL; Ampicillin (Amp), 100 μg/mL; Tetracycline (Tc) 15 μg/mL.

### 2.2 Random Transposon Mutagenesis

The mariner transposon plasmid pALMAR3 was introduced into MOB‐513 as previously described (Malone et al. 2010), and transformants were selected on Lept+Mn agar supplemented with Tc. On this medium, Mn(II)‐oxidising colonies acquire a brown pigmentation while non‐oxidising mutants remain white. Plates were incubated at 28°C for 5 days, and colonies showing either reduced or enhanced Mn(II) oxidation were selected and re‐streaked on fresh Lept+Mn‐Tc plates to confirm the phenotype.

Transposon insertion sites were mapped by arbitrary PCR (O'Toole and Kolter 1998). Primers Arb1b and Arb‐PCR were used for the first amplification round and Arb1 and Almar3‐seq for the second (Malone et al. 2010). Final PCR products were sequenced using the primer Almar3‐seq by Sanger sequencing at the University of Maine. Confirmed mutants were preserved by storage at −70°C of overnight cultures grown in LB supplemented with Tc and mixed with glycerol to a final concentration of 20% (v/v).

### 2.3 Cloning of *rr05389*, *hk05390*, *algR* and *dgc05792* Into pBBR1‐MCS5

The broad‐host‐range vector pBBR1‐MCS5 was used to clone *rr05389*, *hk05390*, *algR*, and *dgc05792* genes (Dombrecht et al. 2001) by the T5 exonuclease‐dependent assembly (TEDA) assembly method (Xia et al. 2019). Each gene was amplified with the primers listed in Table S3, with 20‐bp extensions at their 5′ ends that matched the vector ends generated by SmaI digestion. PCR products were purified using the Zymoclean Gel DNA Recovery Kit (Zymo Research). The pBBR1‐MCS5 vector was linearised with SmaI, gel‐purified, and assembled with each insert using TEDA (Xia et al. 2019). Ligation mixtures were electroporated into *E. coli* and plated on LB agar supplemented with Gm, 0.5 mM IPTG, and 60 μg/mL X‐gal. Positive clones were confirmed by colony PCR and verified by Sanger sequencing at the University of Maine, yielding constructs p*rr05389*, p*hk05390*, p*algR*, and p*dgc05792*.

### 2.4 Mn(II) Oxidation Assays

Mn(II) oxidation was measured as previously described (Piazza et al. 2019). Macrocolonies of each MOB‐513 strain were grown on Lept+Mn agar plates for 4 days at 28°C. Cells were collected from the plate, washed with 10 mM Hepes (pH = 7.5) and resuspended in 1 mL of the same buffer supplemented with 1 mM phenylmethylsulfonyl fluoride (PMSF). Cell lysis was achieved by sonication at 12 mA (1 s pulse and 3 s pause for 20 cycles) and the resulting extracts were standardised by total protein concentration, determined using the Bradford method (Bradford 1976). Mn oxides present in these suspensions were then quantified by the leucoberbelin blue (LBB) assay.

### 2.5 Biofilm Formation Assays

Biofilm biomass was quantified as previously described (Piazza et al. 2019). Briefly, LB cultures at exponential phase (OD_600_ = 0.8) were diluted 1:10 into 2 mL of fresh LB and incubated statically in glass tubes at 28°C for 7 days. Then the medium was carefully removed, and the tubes were gently rinsed three times with distilled water to remove non‐adherent cells. The attached biofilms were stained with Crystal Violet (CV) at 0.3% (w/v) for 15 min at room temperature; unbound dye was discarded and the tubes washed again with distilled water. The CV retained by the biofilm was dissolved into 70% (v/v) ethanol, and the absorbance was measured at 590 nm.

### 2.6 Motility Assays

Twitching motility was assessed by subagar stab inoculation as previously described (Semmler et al. 1999). Briefly, 2 μL of an exponential culture adjusted to OD_600_ = 0.5 was inoculated at the interface between the agar and the plastic of Petri dishes containing 1% (w/v) agar Lept+Mn. Twitching zones were allowed to develop for 24 h at 28°C, after which the agar layer was carefully removed and the halo remaining on the polystyrene was stained with 0.3% (w/v) CV for 5 min. Twitching areas were quantified using ImageJ software (version 1.48) (Schindelin et al. 2012).

Swimming motility was assessed by spotting 5 μL of an overnight culture standardised to OD_600_ = 1.5 at the centre of LB plates solidified with 0.3% (w/v) agar. Migration‐zone diameters were recorded after 24 h at 28°C (Thompson et al. 2023).

### 2.7 Transmission Electron Microscopy (TEM)

MOB‐513 and its derivatives were cultured for 48 h at 28°C on Lept+Mn agar plates. Cells were gently scraped from the agar surface and resuspended in 1× phosphate‐buffered saline (PBS; 137 mM NaCl, 2.7 mM KCl, 10 mM Na_2_HPO4, 1.8 mM KH_2_PO_4_, pH 7.4). Equal volumes of the cell suspension and of 2% (v/v) phosphotungstic acid (H_3_PW_12_O_40_) were mixed, and nickel grids coated with Formvar were floated on the mixture for 1 min. The grids were briefly rinsed with distilled water, the residual liquid was drained with filter paper, and samples were air‐dried for 5 min.

Specimens were examined on a Zeiss LEO906 microscope operated at 80 kV, with images captured on a Megaview G3 digital camera (EMSIS GmbH, Münster, Germany). Imaging was carried out at the electron microscopy facility of the Centro de Microscopía Electrónica (Facultad de Ciencias Médicas, Universidad Nacional de Córdoba, Córdoba, Argentina) at the Instituto de Investigaciones en Ciencias de la Salud (INICSA‐CONICET).

### 2.8 Determination of c‐di‐GMP Intracellular Levels

The c‐di‐GMP biosensor was integrated into the different MOB‐513 strains by co‐electroporation (Choi et al. 2006) of two plasmids: pBGPelA, in which msfGFP (monomeric superfolder GFP) is transcribed from the c‐di‐GMP‐responsive *P*_pelA_ promoter (Martino et al. 2025), and pTnS‐1, supplying the transposase required for chromosomal integration. Recombinant clones carrying the attTn7::[P_pelA_ (BCD2) → *msfGFP*] insertions were selected on LB agar plates supplemented with Sp. Correct chromosomal integration was verified by the loss of Km resistance, which indicates the loss of the non‐integrated pBGPelA plasmid, and was subsequently confirmed by colony PCR (Choi et al. 2005).

Reporter output was read as previously described (Piazza et al. 2022). Briefly, macrocolonies were recovered after 2 days of growth on Lept+Mn agar, washed and resuspended in PBS to an OD_600_ of 0.5. Aliquots of each suspension (200 μL) were dispensed into black, flat‐bottom 96‐well plates (Greiner Bio‐One), and read on a Synergy 2 plate reader (BioTek), recording fluorescence together with OD_600_ through 485 ± 20 nm excitation and 535 ± 20 nm emission filters, respectively.

Normalised fluorescence values (F) were obtained by relativising the OD_600_ and correcting the background signal of the reporter‐free control from that of its reporter‐bearing counterpart, with the formula: F = [RFU(1)/OD_600_(1)] − [RFU(2)/OD_600_(2)], where subscript 1 designates the strain carrying the c‐di‐GMP biosensor, and subscript 2 corresponds to the same strain lacking the biosensor.

### 2.9 Label‐Free Proteomics Analysis

Macrocolony biofilms of the different MOB‐513 strains were grown on Lept+Mn agar plates at 28°C for 2 days (four biological replicates per strain) and cells were harvested from the agar surface, resuspended in PBS, and processed for whole‐cell protein extraction as previously described (Piazza et al. 2022). After protein quantification using the Bicinchoninic Acid (BCA) assay (Walker 1994), aliquots containing 30 μg of total protein were submitted to the Unidad de Espectrometría de Masa (IBR‐CONICET) for LC–MS analysis.

### 2.10 LC–MS Analysis

Peptide separations were conducted using an Ultimate3000 nanoHPLC system (Thermo Scientific) with a nano column EASY‐Spray ES903 (50 cm × 50 μm ID, PepMap RSLC C18). The mobile phase flow rate was 300 nL/min using 0.1% (v/v) formic acid in water (Solvent A). and 0.1% formic acid in acetonitrile (Solvent B), with a gradient profile of 4%–30% solvent B for 114 min, 30%–80% solvent B for 14 min, and 80% solvent B for 2 min. Subsequently, MS analysis was performed on a Q‐Exactive HF mass spectrometer (Thermo Scientific). 1.9 kV of liquid‐junction voltage and 250°C of capillary temperature were used for ionisation. The parameters for the full scan method were m/z 375–2000 mass selection, an Orbitrap resolution of 120,000 (at m/z 200), a maximum injection time of 100 ms and a target automatic gain control (AGC) value of 1 e^6^.

After the survey scan we selected the 8 most intense precursor ions for MS/MS fragmentation, which was performed with a normalised collision energy of 27 eV. MS/MS scans were acquired with a dynamic first mass. The resolution was 30,000 (at m/z 200), AGC target was 5 e^5^, intensity threshold was 1.5 e^5^, isolation window was 1.4 m/z, and maximum injection time was 55 ms. We enabled charge‐state screening to reject unassigned, singly charged, and equal to or more than six protonated ions. A dynamic exclusion period of 30 s was used to prevent repeated selection of previously fragmented precursor ions.

### 2.11 MS Data Analysis

MS data were analysed using MaxQuant (V: 2.5.1.0). with standardised workflows. We searched mass spectra *.raw files against a database from *P. resinovorans* . Precursor and fragment mass tolerance were set to 10 ppm and 0.02 Da, respectively, allowing 2 missed cleavages. The following fixed modification was included: carbamidomethylation of cysteines. The following variable modification was included: protein N‐terminal acetylation and methionine oxidation.

Relative protein abundances were quantified using the label‐free quantification algorithm and the resulting matrix was processed in Perseus (Tyanova et al. 2016). A protein was considered differentially abundant when the mean normalised difference between conditions was ≥ 2.5 or ≤ −2.5 with a *p* value ≤ 0.05.

### 2.12 Identification of Two‐Component Systems in MOB‐513

Two‐component system (TCS) proteins encoded in the MOB‐513 genome were analysed using the Predicted Prokaryotic Regulatory Proteins (P2RP) software (Barakat et al. 2013). A total of 60 histidine kinases (HKs) and 80 response regulators (RRs) were identified based on reciprocal BLASTn/p analyses. HKs were classified according to their domain architecture as either classical or hybrid kinases, while RRs were categorised based on the type of effector domain present, including DNA‐binding (e.g., NarL, OmpR, LytTR), RNA‐binding (AmiR), enzymatic (GGDEF, EAL), or the absence of an effector domain (REC‐only).

### 2.13 Statistical Analysis

All quantifications were performed from three biological replicates; each assayed in technical triplicates, and all graphs display mean values with their standard deviations. Statistical analyses were performed using GraphPad Prism (Version 8) by applying one‐way ANOVA followed by Tukey's post hoc test (*p* < 0.05).

Asterisks in the figures indicate statistically significant differences between the compared strains, with exact *p* value thresholds detailed in each figure legend.

## 3 Results

### 3.1 Random Transposon Mutagenesis Identifies Key Factors Driving Both Loss and Enhancement of Mn(II) Oxidation in MOB‐513

To investigate the genetic determinants of Mn(II) oxidation, we undertook a random mutagenesis screen in MOB‐513 strain. This strain, originally isolated from a biological treatment plant (Piazza et al. 2019), produces biogenic MnOx that precipitate on the cell surface after 3 days of growth under static conditions on Mn(II)‐containing selective medium (Lept+Mn). Under these conditions, MOB‐513 develops a characteristic brown coloration resulting from MnOx accumulation, providing a convenient visual marker for screening.

A random transposon library comprising ~2000 clones was screened on Lept+Mn agar plates to identify mutants with altered Mn(II)‐oxidation phenotypes, characterised by either loss of oxidation (cream‐coloured colonies) or enhanced oxidation (dark brown) phenotypes. From this screen, 80 non‐oxidising and 18 hyper‐oxidising mutants were selected for sequencing and genomic mapping (Table 1).

**TABLE 1:** Genes disrupted in MOB‐513 Mn(II)‐oxidation mutants identified by transposon mutagenesis.

Locus tag	Phenotype	Protein name	Predicted function	*N* ^o^ of insertions
*PRES513_01032* (*pilC*)	Non‐oxidising	Tfp assembly protein PilC	Tfp Biogenesis or assembly	10
*PRES513_01032*	Non‐oxidising	Tfp assembly, ATPase PilB	Tfp Biogenesis or assembly	8
*PRES513_01031*	Non‐oxidising	Prepilin peptidase PilD	Tfp Biogenesis or assembly	5
*PRES513_05901*	Non‐oxidising	Tfp biogenesis protein PilM	Tfp Biogenesis or assembly	4
*PRES513_01211*	Non‐oxidising	Tfp biogenesis protein PilF	Tfp Biogenesis or assembly	2
*PRES513_00994*	Non‐oxidising	Tfp biogenesis protein PilE	Tfp Biogenesis or assembly	2
*PRES513_05898*	Non‐oxidising	Tfp biogenesis protein PilP	Tfp Biogenesis or assembly	2
*PRES513_05897*	Non‐oxidising	Tfp biogenesis protein PilQ	Tfp Biogenesis or assembly	2
*PRES513_00293*	Non‐oxidising	Twitching motility protein PilT	Tfp Biogenesis or assembly	2
*PRES513_05900*	Non‐oxidising	Tfp biogenesis protein PilN	Tfp Biogenesis or assembly	1
*PRES513_05899*	Non‐oxidising	Tfp biogenesis protein PilO	Tfp Biogenesis or assembly	1
*PRES513_05390*	Non‐oxidising	Histidine Kinase, HK05390	c‐di‐GMP Metabolism	4
*PRES513_01015*	Non‐oxidising	Hypothetical Protein (With a PilZ domain protein)	c‐di‐GMP Metabolism	4
*PRES513_00551*	Non‐oxidising	Bis(5′‐nucleosyl)‐tetraphosphatase	c‐di‐GMP Metabolism	2
*PRES513_06158*	Non‐oxidising	Two‐component system response regulator, AlgR	Biofilm Formation	2
*PRES513_01588*	Non‐oxidising	Carbon storage regulator	Biofilm Formation	2
*PRES513_04472*	Non‐oxidising	ATP‐dependent Clp protease proteolytic subunit	Biofilm Formation	1
*PRES513_01552*	Non‐oxidising	L‐aspartate oxidase (EC 1.4.3.16)	Cofactor Biosynthesis	5
*PRES513_00369*	Non‐oxidising	Hypothetical protein (YccS family putative transporter)	Transmembrane Transport	8
*PRES513_00174*	Non‐oxidising	Outer membrane low permeability porin, OprD family	Transmembrane Transport	1
*PRES513_01215*	Non‐oxidising	Putative transporter	Transmembrane Transport	2
*PRES513_01595*	Non‐oxidising	Ribose 1,5‐bisphosphate phosphokinase PhnN	Phosphate Transport and Metabolism	1
*PRES513_06248*	Non‐oxidising	Phosphate transport system permease protein PstC	Phosphate Transport and Metabolism	1
*PRES513_06241*	Non‐oxidising	Phosphate regulon sensor protein PhoR (SphS)	Phosphate Transport and Metabolism	1
*PRES513_01286*	Non‐oxidising	Methyl‐accepting chemotaxis sensor/transducer protein	Amino Acid Chemotaxis, Metabolism and Transport	1
*PRES513_01596*	Non‐oxidising	Transcriptional regulator, AsnC family	Amino Acid Chemotaxis, Metabolism and Transport	1
*PRES513_00264*	Non‐oxidising	Probable coniferyl aldehyde dehydrogenase	Aromatic Compounds Metabolism	1
*PRES513_04568*	Non‐oxidising	3‐Oxoadipate enol‐lactonase, alpha/beta hydrolase fold family	Aromatic Compounds Metabolism	1
*PRES513_05177*	Non‐oxidising	Methionine aminopeptidase	Protein Translation and Maturation	1
*PRES513_04516*	Non‐oxidising	Alpha‐L‐glutamate ligase family protein	Protein Translation and Maturation	1
*PRES513_02917*	Non‐oxidising	Excinuclease ABC subunit C	Nucleotide‐excision Repair	1
*PRES513_05792*	Hyper‐oxidising	Diguanylate cyclase, Dgc05792	c‐di‐GMP Biosynthesis, Metabolism or Response	10
*PRES513_05573*	Hyper‐oxidising	Signal transduction hybrid histidine kinase, HK05573	Biofilm Formation	2
*PRES513_05741*	Hyper‐oxidising	Signal transduction hybrid histidine kinase, HK05741	Biofilm Formation	1
*PRES513_02938*	Hyper‐oxidising	Transcriptional regulator, AraC family	Transcriptional Regulation	2
*PRES513_00288*	Hyper‐oxidising	Putative dynamin‐like GTPase family protein	Membrane‐Related Processes	2
*PRES513_02910*	Hyper‐oxidising	Integrase	DNA Integration and Recombination	1

*Note:* Locus tags, protein names, predicted protein functions and the number of independent transposon insertions are shown for mutants displaying either non‐oxidising or hyper‐oxidising phenotypes.

Among the non‐oxidising mutants, 39 (48.75%) carried transposon insertions in genes encoding proteins involved in Tfp biogenesis or assembly, spanning 11 distinct genes. This strong enrichment suggests that Tfp plays an essential role in the Mn(II) oxidation process. Notably, *pilC*, which encodes a core component of Tfp biogenesis machinery (Takhar et al. 2013), was the most frequently disrupted gene, with 10 independent insertions. Consistent with this, two insertions disrupted *PRES513_06158*, encoding a two‐component response regulator, homologous to AlgR (90.7% identity to *P. aeruginosa* AlgR). In *P. aeruginosa* , AlgR directly activates transcription of the *fimU–pilVWXY1Y2E* operon, which encodes minor pilins essential for Tfp biogenesis and function, promotes biofilm formation, and modulates swimming motility (Okkotsu et al. 2014; Belete et al. 2008).

Transmembrane transport genes were the second most affected category, with 11 non‐oxidising mutants (13.75%) carrying insertions in four different transporters. This suggests that the non‐oxidising phenotype may result from impaired secretion of the Mn(II)‐oxidising factors.

Insertions in genes involved in c‐di‐GMP metabolism or signalling accounted for 10 non‐oxidising mutants (12.5%), while biofilm‐related genes were disrupted in 5 mutants (6.25%), supporting a critical role for biofilm formation in MOB‐513‐Mn(II) oxidation (Piazza et al. 2022). Four of the c‐di‐GMP‐related insertions mapped to *PRES513_01015*, encoding a PilZ‐domain protein, previously shown to link c‐di‐GMP signalling and Mn(II) oxidation in MOB‐513 (Piazza et al. 2022), while two affected the Bis(5′‐nucleosyl)‐tetraphosphatase gene (*PRES513_00551*), suggesting that nucleotide‐pool regulation may indirectly influence c‐di‐GMP levels (Monds et al. 2010).

Four insertions were identified in the gene *PRES513_05390*, predicted to encode a multidomain histidine kinase and here designated HK05390, which homology searches and phylogenetic analysis placed within a distinct clade of uncharacterised histidine kinases in *Pseudomonas*. Interestingly, *hk05390* is genomically adjacent to *PRES513_05389*, which encodes a GGDEF/EAL‐domain response regulator, here designated RR05389. We therefore hypothesised that HK05390 and RR05389 constitute a cognate two‐component regulatory system modulating intracellular c‐di‐GMP levels.

Smaller groups of mutants pointed to additional cellular processes potentially involved in Mn(II) oxidation, including L‐aspartate oxidase (three non‐oxidising mutants, 3.75%), genes involved in phosphate transport and metabolism (three mutants, 3.75%), amino acid chemotaxis, metabolism, and transport; protein translation and maturation (two mutants, 2.50%), aromatic compound metabolism (two mutants, 2.50%), and a single mutation in a nucleotide‐excision repair gene (one mutant, 1.25%).

Among the 18 hyper‐oxidising clones, 10 (55.56%) carried transposon insertions in the promoter region of *PRES513_05792*, which encodes a GGDEF‐domain protein likely involved in c‐di‐GMP biosynthesis, here designated Dgc05792. Given the increased Mn(II) oxidation observed upon overexpression of the *B. pertussis* *dgcB* gene in MOB‐513 (Piazza et al. 2022), we speculated that the transposon insertions located in the promoter region of *dgc05792* were driving its expression, leading to increased intracellular c‐di‐GMP levels and thereby enhanced Mn(II) oxidation.

Three mutants (16.70%) had insertions disrupting hybrid sensor histidine kinases. Two independent mutants contained insertions in *PRES513_05573*, here designated as HK05573 and one mutant in *PRES513_05741*, here designated as HK05741. Additionally, mutations in AraC‐family transcriptional regulators were found in two clones (11.10%) (PRES513_02938), highlighting potential roles for two‐component systems (TCS) and transcriptional regulation in environmental sensing and regulation of oxidation pathways (Stock et al. 2000). Finally, two mutants (11.10%) were disrupted in genes encoding dynamin‐like GTPases, and one mutant (5.60%) carried an insertion in an integrase gene.

### 3.2 Functional Insights Into Mn‐Regulatory Two‐Component System Elements in MOB‐513

Since several transposon mutants disrupted putative regulatory proteins, we performed a predictive analysis to identify two‐component systems (TCSs) (Barakat et al. 2013) in the MOB‐513 genome. This analysis revealed many TCSs (Table S4), specifically 60 histidine kinases (HK) and 80 response regulators (RR). Notably, *hk05390* and *rr05389*, 3729 and 2163 nucleotides in length, respectively, overlap by 19 nucleotides and are likely co‐transcribed, suggesting a coordinated regulatory function.

Additionally, P2RP predicts that AlgR functions as a RR containing a LytTR DNA‐binding domain, and it is likely paired with the upstream histidine kinase PRES513_06159 (AlgZ), forming another TCS associated with Mn(II) oxidation (Table S4). We hypothesise that this system may influence Tfp deployment/production in MOB‐513, given the high number of pili genes linked to non‐oxidising mutations. P2RP analysis also confirmed that both *hk05573* and *hk05741* genes encode hybrid sensor HKs (Table S4).

In *P. putida* GB‐1, the MnxS1‐MnxS2‐MnxR regulatory pathway, composed of two tandem sensor HKs and a cognate RR (PputGB1_2519, PputGB1_2520 and PputGB1_2521), has been shown to be essential for Mn(II)‐oxidation (Geszvain and Tebo 2010). Therefore, we performed BLASTP analyses to evaluate the similarity between the MOB‐513 TCSs identified in the transposon screen and the MnxS1‐MnxS2‐MnxR system (Table S5). Overall, none of the MOB‐513 regulators showed significant similarity to the MnxS1‐MnxS2‐MnxR system, suggesting that Mn‐oxidation in MOB‐513 is regulated by a distinct two‐component regulatory network (Table S5).

Among the MOB‐513 proteins, HK05390 and HK05573 showed moderate to weak similarity to the multi‐sensor hybrid histidine kinase PputGB1_2142 in GB‐1 (39.19% and 30.77% identity, respectively). HK05741 displayed higher similarity (65.56% identity) to an integral membrane sensor histidine kinase (PputGB1_4877). RR05389 showed strong similarity (58.75% identity) to a PAS/PAC domain‐containing regulatory protein associated with diguanylate cyclase/phosphodiesterase activity (PputGB1_5008). AlgR was highly conserved (79.84% identity) with its GB‐1 homologue (PputGB1_0210), whereas its putative cognate kinase AlgZ showed no detectable similarity to GB‐1 proteins.

### 3.3 Loss of Mn(II) Oxidation Correlates With Reduced Biofilm Formation in MOB‐513 Transposon Mutants

Given that the largest fraction of insertions mapped to genes involved in Tfp biogenesis (Table 1), we focused on the two non‐oxidising mutants: *pilC* and *algR*. We further examined the two mutants with potential effects on c‐di‐GMP metabolism or regulation (Table 1). The first was the non‐oxidising mutant disrupted in *hk05390*, the uncharacterised histidine kinase potentially associated with the adjacent GGDEF/EAL‐domain gene *rr05389*. The second mutant showed a hyper‐oxidising phenotype with a transposon insertion in the promoter region of the gene encoding the putative diguanylate cyclase, Dgc05792.

Most transposon insertions that disrupted *pilC*, *algR*, *hk05390*, and the *dgc05792* promoter occurred close to each other within a small region of each gene. Therefore, one representative clone per mutant was selected. The non‐oxidising mutants were designated as MOB‐513‐*pilC*::Tn, MOB‐513‐*algR*::Tn, and MOB‐513‐*hk05390*::Tn and the hyper‐oxidising strain was named MOB‐513‐*dgc05792p*::Tn. The reduced abundance of PilC, AlgR and HK05390 was confirmed by proteomics comparisons between the non‐oxidising mutants and the wild type (WT) strain (Tables S6–S8 and Figures S2–S4). The mutant strains were complemented *in trans*, generating MOB‐513‐*algR*::Tnc and MOB‐513‐*hk05390*::Tnc for subsequent analyses. The non‐oxidising phenotype of MOB‐513‐*pilC*::Tn could not be restored by complementation *in trans*, consistent with polar effects within the *pil* operon. However, given that numerous independent transposon insertions disrupting different Tfp biogenesis genes also abolished Mn(II) oxidation (Table 1), this likely reflects a broader requirement for an intact Tfp apparatus rather than a specific role of PilC alone in Mn(II) oxidation. Conversely, in the hyper‐oxidising MOB‐513‐*dgc05792p*::Tn strain, the abundance of Dgc05792 was increased relative to wild type (Table S9 and Figure S5A,B), consistent with transcriptional upregulation from the outward‐facing transposon insertion at the promoter region (Piazza et al. 2022).

As expected, Mn(II) oxidation assays revealed no detectable MnOx production in the mutants: MOB‐513‐*pilC*::Tn, MOB‐513‐*algR*::Tn, and MOB‐513‐*hk05390*::Tn. The complemented strains MOB‐513‐*algR*::Tnc and MOB‐513‐*hk05390*::Tnc restored the Mn(II) oxidising phenotype to wild‐type levels. In contrast, the hyper‐oxidising strain MOB‐513‐*dgc05792p*::Tn showed a significant 1.96‐fold increase in Mn(II) oxidation relative to the wild type (Figure 1A).

**FIGURE 1:**
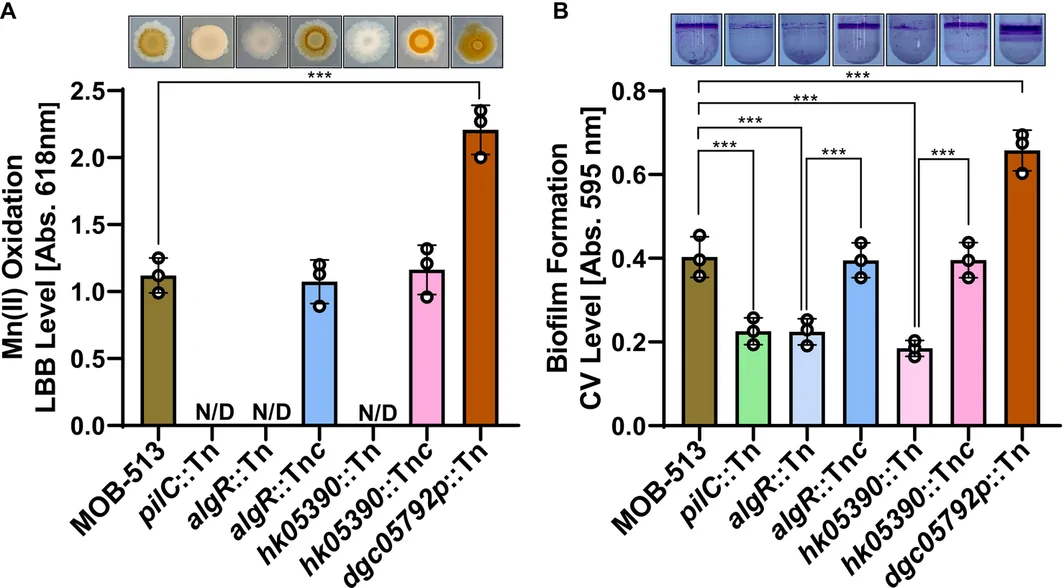
Mn(II) oxidation and biofilm formation in MOB‐513 wild type, Tn‐mutants, and complemented strains. Quantitative assays were conducted for MOB‐513 wild type, the non‐oxidising mutants MOB‐513*‐pilC::Tn* (*pilC::Tn*), MOB‐513*‐algR::Tn* (*algR::Tn*), MOB‐513*‐hk05390::Tn* (*hk05390::Tn*); the hyper‐oxidising mutant MOB‐513*‐dgc05792p::Tn* (*dgc05792p::Tn*); and the complemented strains *algR::Tnc* and *hk05390::Tnc*. (A) Mn(II) oxidation was quantified in macrocolonies grown on Lept+Mn for 4 days by measuring absorbance at 618 nm following leucoberbelin blue (LBB) staining, which reflects Mn(III/IV) oxide accumulation. (B) Biofilm biomass after 72 h of growth in LB at 28°C was quantified at the air‐liquid interface by Crystal Violet (CV) staining and measured spectrophotometrically at 595 nm (Abs. 595 nm). Upper panels display representative images of the observed phenotypes. Values represent the mean of biological triplicates, from three independent experiments; error bars indicate standard deviation. Statistical significance was determined using one‐way ANOVA followed by Tukey's post hoc test. Asterisks indicate significance levels, comparing the mutant with the wild type or their complemented strains: ***, *p* < 0.001. N/D, not detected.

Biofilm formation was significantly reduced in the non‐oxidising mutants, with MOB‐513‐*pilC*::Tn, MOB‐513‐*algR*::Tn, and MOB‐513‐*hk05390*::Tn forming 1.78‐, 1.80‐ and 2.18‐fold less biofilm than the wild‐type strain, respectively (Figure 1B). Complementation with *algR* and *hk05390 in trans* restored biofilm formation (Figure 1B). Conversely, MOB‐513‐*dgc05792p*::Tn exhibited a ~1.63‐fold increase in biofilm formation compared to the wild type (Figure 1B). Together, these results demonstrate a strong correlation between loss of Mn(II)‐oxidising capacity and reduced biofilm formation.

### 3.4 Twitching and Flagellar Swimming Are Differentially Affected in MOB‐513 Mutants

Given that twitching motility depends on Tfp (Jin et al. 2011), and flagellum‐driven swimming often inversely correlates with biofilm formation (Guttenplan and Kearns 2013), both motility types were quantified in the mutants.

Twitching motility was abolished in MOB‐513‐*pilC*::Tn and MOB‐513‐*algR*::Tn, and was impaired in MOB‐513‐*hk05390*::Tn, which exhibited 1.90‐fold reduction relative to the wild type. *In trans* complementation, restored twitching in both MOB‐513‐*algR*::Tn and MOB‐513‐*hk05390*::Tn (Figure 2A). MOB‐513‐*dgc05792p*::Tn showed twitching levels similar to the wild type (Figure 2A).

**FIGURE 2:**
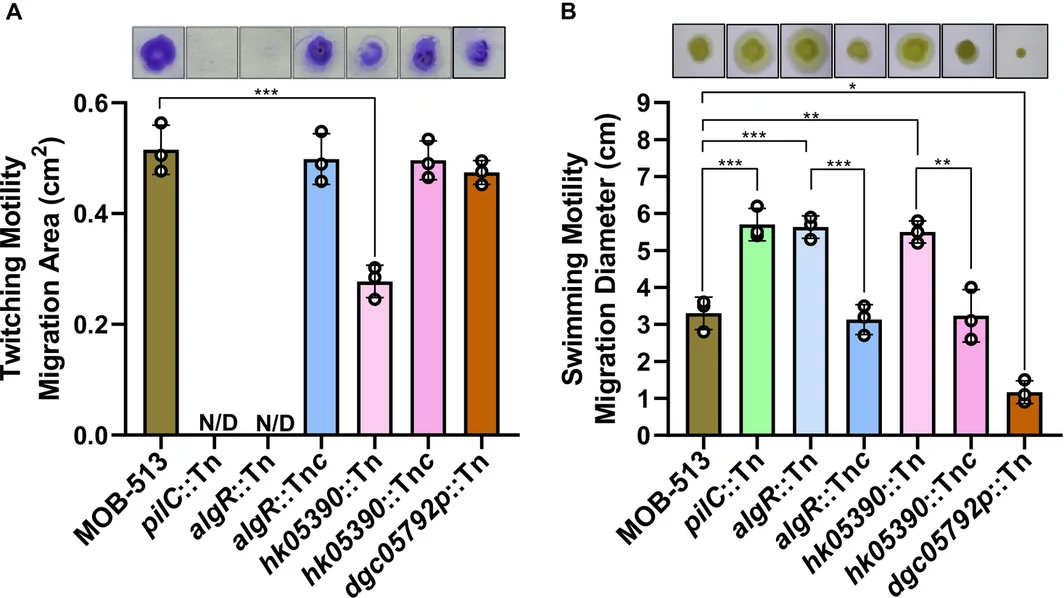
Motility assays in MOB‐513 wild type, Tn‐mutants and complemented strains. Quantitative assays were conducted for the strains mentioned in Figure 1. (A) Twitching motility was evaluated by measuring the area (cm^2^) of interstitial migration at the LB agar‐polystyrene interface after 24 h of incubation at 28°C. (B) Swimming motility was assessed by measuring the diameter (cm) of radial migration through 0.3% LB agar after 24 h at 28°C. Upper panels display representative images of the observed phenotypes. Values represent the mean of biological triplicates, from three independent experiments, error bars indicate standard deviation. Statistical significance was determined using one‐way ANOVA followed by Tukey's post hoc test. Asterisks indicate significance levels, comparing the mutant with the wild type or their complemented strains: *p < 0.05; **p < 0.01; ***p < 0.001. N/D, not detected.

Next, soft‐agar plates were used to examine the swimming mode of growth. As anticipated, the colony diameter was enhanced in the non‐oxidising strains (Figure 2B), consistent with previous results (Piazza et al. 2022). MOB‐513‐*pilC*::Tn, MOB‐513‐*algR*::Tn, and MOB‐513‐*hk05390*::Tn migrated 1.72‐, 1.70‐, and 1.67‐fold more than the wild type, respectively, and complementation restored these phenotypes to a level similar to the wild type, in terms of colony diameter (Figure 2B). In contrast, MOB‐513‐*dgc05792p*::Tn, which exhibited increased biofilm formation (Figure 1B), displayed a strong reduction in swimming motility, migrating 2.82‐fold less than the wild type (Figure 2B).

Next, transmission electron microscopy (TEM, 7000–40,000×) was used to establish whether any defect existed in the assembly of the flagella and Tfp as a result of transposon insertions in these mutants. The electron micrographs revealed two distinct cell subpopulations in the wild type, one densely covered with numerous thin filaments surrounding the cell surface, consistent with Tfp production (Lindhout et al. 2009) (Figure 3A), and a second exhibiting a polar flagellar filament and fewer pili (Figure 3B). This observation is interesting considering our previous studies of macrocolony biofilms, which show that MOB‐513 biofilms consist of two distinct subpopulations of cells, with Mn(II) oxidisers located at the top and non‐oxidisers at the bottom (Piazza et al. 2022). While the functional roles of the morphologically distinct cells observed here remain to be determined, these differences may correspond to the spatially and functionally distinct subpopulations described in biofilms.

**FIGURE 3:**
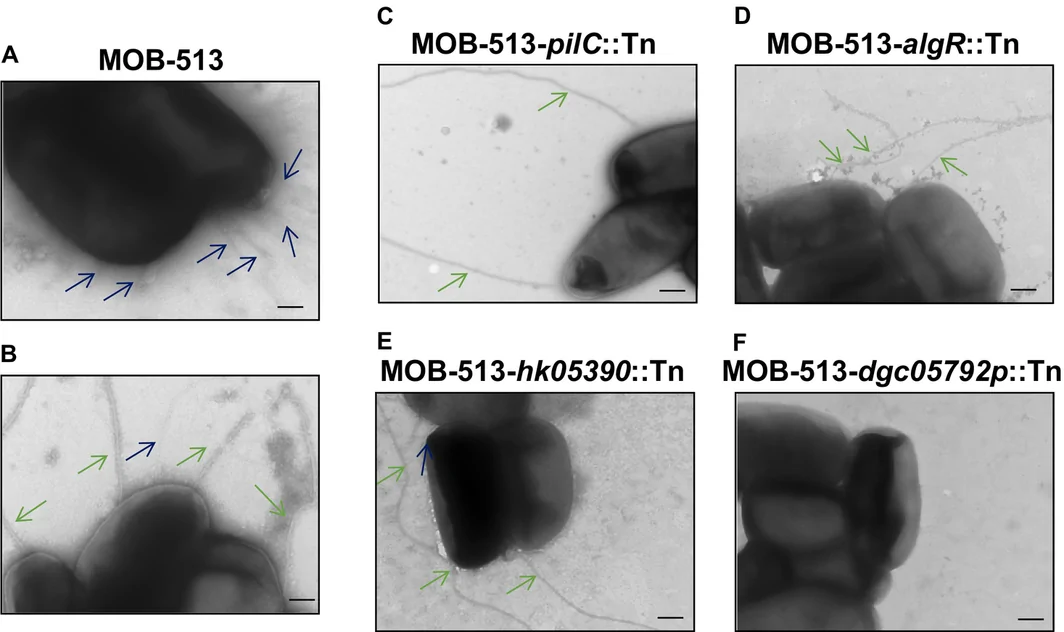
Transmission electron microscopy detection of appendages on the surface of MOB‐513 wild type and Tn‐mutants. Negative‐stain TEM images (7000–40,000×) used to visualise assembled Type IV pili (indicated by blue arrows) and flagella (indicated by green arrows) in MOB‐513 wild type (A, B), MOB‐513‐*pilC*::Tn (C), MOB‐513‐*algR*::Tn (D), MOB‐513‐*hk05390*::Tn (E), and MOB‐513‐*dgc05792p*::Tn (F), grown in Lept+Mn for 48 h. Scale bar: 200 nm.

Consistent with the twitching results, MOB‐513‐*pilC*::Tn and MOB‐513‐*algR*::Tn lacked detectable pili, whereas MOB‐513‐*hk05390*::Tn showed fewer pili than the wild type (Figure 3C–E). Interestingly, MOB‐513‐*dgc05792p*::Tn exhibited no detectable pili (Figure 3F), although twitching data (Figure 2A) suggest that pili may form transiently at earlier growth stages. We previously showed that Mn(II) oxidation is accelerated and enhanced in strains with increased c‐di‐GMP levels (Piazza et al. 2022), suggesting that the determinants contributing to oxidation may be activated earlier under these conditions.

Together, these results confirm the importance of PilC for Tfp formation and suggest positive regulatory roles for AlgR and HK05390 in the biogenesis of these structures in MOB‐513.

Consistent with the swimming assays, the electron micrographs showed MOB‐513‐*pilC*::Tn, MOB‐513‐*algR*::Tn, and MOB‐513‐*hk05390*::Tn all assemble flagellar structures on the surface of the cell, whereas MOB‐513‐*dgc05792p*::Tn lacked detectable flagella (Figure 3C–F). These results corroborate the inverse relationship between swimming and biofilm formation/Mn(II)‐oxidation in the mutant strains.

### 3.5 Altered c‐di‐GMP Levels in MOB‐513 Mutants Lead to Different Outcomes

Intracellular c‐di‐GMP levels in MOB‐513 WT and the mutant strains were assessed using a fluorescent msfGFP‐based reporter system driven by the *P. aeruginosa* c‐di‐GMP‐responsive *P*_*pelA*_ promoter (Baraquet et al. 2012; Benedetti et al. 2016). To functionally validate the biosensor in the MOB‐513 background, fluorescence was quantified in strains overproducing the *B. pertussis* diguanylate cyclase DgcB (MOB‐513‐p*dgcB*) and the phosphodiesterase PdeA (MOB‐513‐p*pdeA*) (Sisti et al. 2013). As expected, overexpression of *dgcB* resulted in a 1.26‐fold increase in fluorescence compared to the MOB‐513 wild type, while phosphodiesterase *pdeA* overexpression led to a 1.41‐fold decrease (Figure S1A). These results are consistent with previous results (Piazza et al. 2022), validating the use of this reporter system for detecting intracellular c‐di‐GMP in MOB‐513.

The non‐oxidising strains MOB‐513‐*pilC*::Tn, MOB‐513‐*algR*::Tn, and MOB‐513‐*hk05390*::Tn exhibited a significant decreased fluorescence compared to MOB‐513 wild type, with 1.55‐, 1.68‐ and 1.51‐fold decrease, respectively. In contrast, the MOB‐513‐*dgc05792p*::Tn mutant showed a significant 1.30‐fold increase in fluorescence, consistent with c‐di‐GMP accumulation (Figure 4A).

**FIGURE 4:**
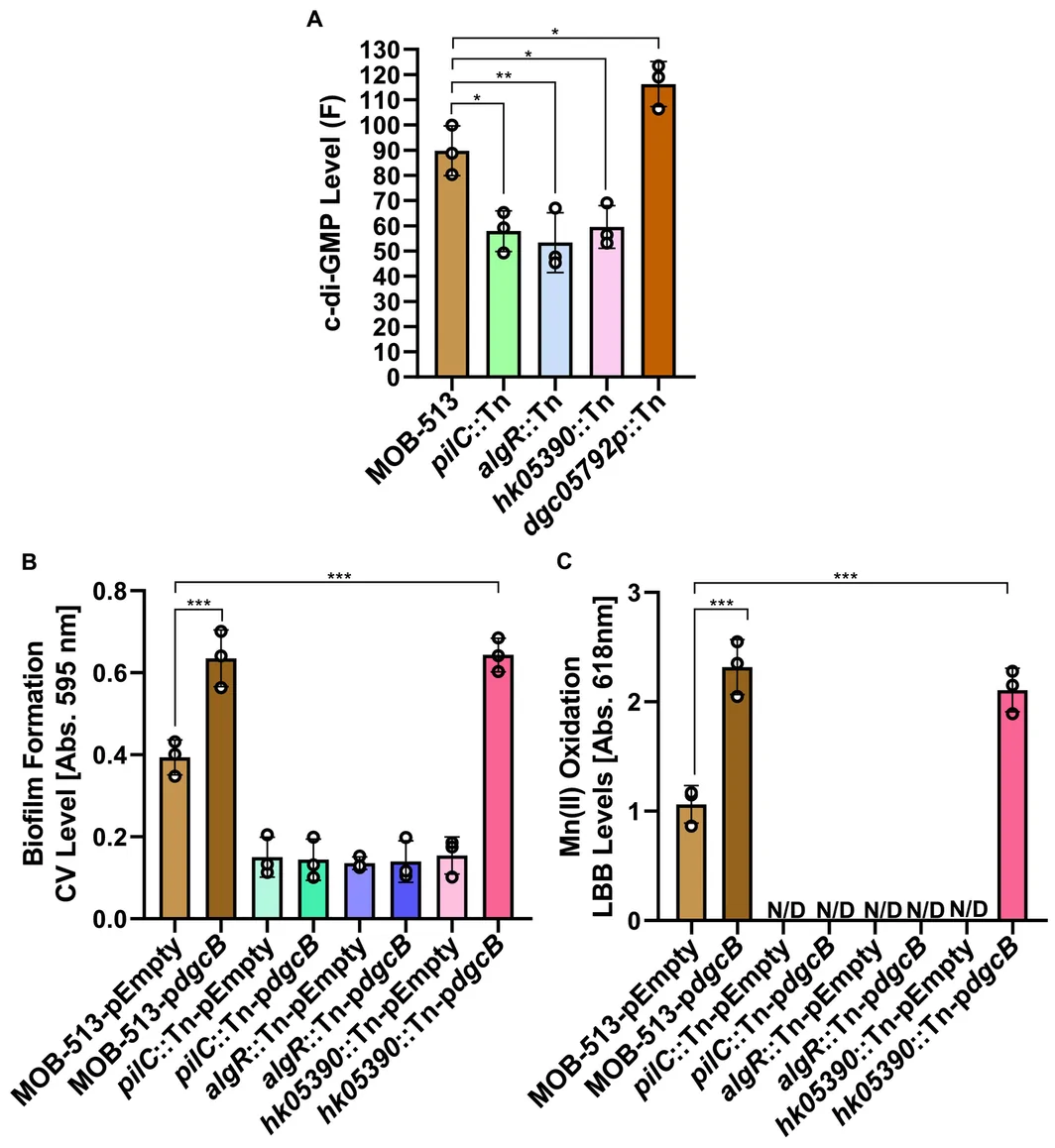
Intracellular c‐di‐GMP levels, biofilm formation, and Mn(II) oxidation in MOB‐513 wild type and Tn‐mutant strains. (A) Intracellular c‐di‐GMP levels were quantified using the transcriptional biosensor (P_pel_A–msfGFP). Macrocolonies of MOB‐513 WT, *pilC*::Tn, *algR*::Tn, *hk05390*::Tn and *dgc05792p*::Tn were grown on Lept+Mn agar for 2 days. Fluorescence values were normalised to OD_600_, and background fluorescence from biosensor‐free controls was subtracted. (B) Biofilm formation and (C) Mn(II)‐oxidation were quantified as described in Figure 1 for MOB‐513‐pEmpty, MOB‐513‐p*dgcB*, and the corresponding pEmpty or p*dgcB*‐transformed mutants. Values represent the mean of biological triplicates, from three independent experiments, error bars indicate standard deviation. Statistical significance was determined by one‐way ANOVA with Tukey's post hoc test. Asterisks indicate significance levels, comparing the wild type with the mutant strains (A) or pEmpty‐transformed strains compared with p*dgcB*‐transformed strains (B and C): * *p* < 0.05; **p < 0.01; ***p < 0.001. N/D, not detected.

Then, we investigated whether the loss of Mn(II) oxidation and biofilm formation in MOB‐513‐*pilC*::Tn, MOB‐513‐*algR*::Tn, and MOB‐513‐*hk05390*::Tn mutants could be restored by increased c‐di‐GMP levels. Therefore, we transformed these strains with the plasmid p*dgcB* (Piazza et al. 2022; Sisti et al. 2013), to overproduce DgcB and elevate intracellular c‐di‐GMP levels.

The resulting strains, MOB‐513‐*pilC*::Tn‐p*dgcB*, MOB‐513‐*algR*::Tn‐d*gcB*, and MOB‐513*‐hk05390*::Tn‐p*dgcB*, displayed intracellular c‐di‐GMP levels similar to MOB‐513p*dgcB* (Figure S1B), restoring the defects observed in the mutants (Figure 4A).

Increased c‐di‐GMP levels in the non‐oxidiser MOB‐513‐*hk05390*::Tn mutant fully restored Mn(II) oxidation and biofilm formation phenotypes (Figure 4C) but had no impact on the strains MOB‐513‐*pilC*::Tn and MOB‐513‐*algR*::Tn (Figure 4B,C). Together, these results suggest that HK05390 functions upstream of a diguanylate cyclase to regulate c‐di‐GMP homeostasis, directly implicated in Mn(II) oxidation, while also showing that the complete loss of pili cannot be compensated by increasing c‐di‐GMP levels.

### 3.6 Dgc05792 and RR05389 Elevates c‐di‐GMP and Promotes Mn(II) Oxidation and Biofilm Formation

Since Dgc05792 and RR05389 remain uncharacterised, an alignment of their amino acid sequences with well‐characterised DGCs and PDEs was performed. The presence of a conserved GGDEF in both proteins, and a conserved EAL domain in RR05389, was observed (data not shown). Given that the canonical GG[D/E]EF domain is present in both proteins, they would be predicted to have DGC activity (Jenal and Malone 2006). Therefore, to assess their potential roles in c‐di‐GMP metabolism, the wild‐type strain was transformed with plasmids constitutively expressing *dgc05792* (MOB‐513‐p*dgc05792*) or *rr05389* (MOB‐513‐p*rr05389*). Both strains exhibited elevated c‐di‐GMP reporter signals (Figure S1B). MOB‐513‐pEmpty and MOB‐513‐p*dgcB* served as controls.

Both MOB‐513‐pdgc05792 and MOB‐513‐p*rr05389* showed increased biofilm formation and Mn(II) oxidation relative to MOB‐513‐pEmpty, 1.68‐ and 1.80‐fold for p*dgc05792*, and 2.43‐ and 2.62‐fold for p*rr05389*, respectively (Figure 5A,B). These results suggest that Dgc05792 and RR05389 increase intracellular c‐di‐GMP levels, thereby promoting biofilm formation and Mn(II) oxidation.

**FIGURE 5:**
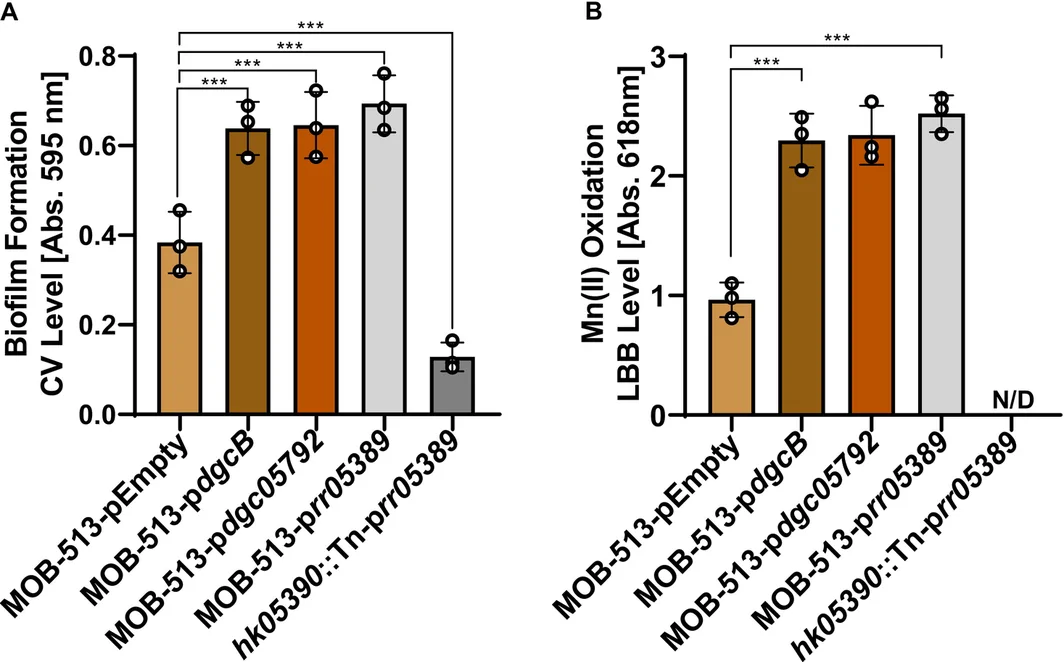
Functional analysis of Dgc05792 and RR05389 in biofilm formation and Mn(II) oxidation. (A) Biofilm formation and (B) Mn(II)‐oxidation were quantified as in Figure 1 in MOB‐513 strains overexpressing the diguanylate cyclase DgcB (positive control) or Dgc05792, and the response regulator RR05389. RR05389 was also overexpressed in the *hk05390*::Tn mutant background. Values represent the mean of biological triplicates, from three independent experiments, error bars indicate standard deviation. Statistical significance was determined using one‐way ANOVA followed by Tukey's post hoc test. Asterisks denote significant differences of p*Empty*‐transformed strains compared with p*dgc05792*‐transformed or p*rr05389*‐transformed strains: ***p < 0.001. N/D, not detected.

Furthermore, RR05389 dependence on HK05390 was evaluated by overexpressing *rr05389* in the *hk05390* mutant background (MOB‐513‐*hk05390*::Tn‐p*rr05389*), which failed to restore biofilm formation (Figure 5A) and Mn(II) oxidation phenotypes (Figure 5B). These findings suggest that RR05389 requires HK05390 for its functional output on c‐di‐GMP‐regulated phenotypes, consistent with the possibility that they operate as a cognate two‐component pair modulating intracellular c‐di‐GMP levels.

### 3.7 Non‐Oxidising and Hyper‐Oxidising Mutants Exhibit Specific Proteomic Changes Relative to the MOB‐513 Wild Type

To investigate the physiological changes associated with transposon insertions in MOB‐513, we conducted a comparative proteomic analysis between MOB‐513 WT and MOB‐513‐*pilC*::Tn; MOB‐513‐*algR*::Tn; MOB‐513‐*hk05390*::Tn; and MOB‐513‐*dgc05792*::Tn.

We determined the COG functional categories of MOB‐513 proteins whose abundances were altered by the insertion of the transposon (Figures S2–S5). Overall, the differences between MOB‐513 WT and transposon mutants were limited, suggesting the impact of the transposon insertion on the Mn‐oxidation was relatively specific (Tables S6–S9 and Figures S2–S5).

In MOB‐513‐*pilC*::Tn, PilC was significantly decreased relative to MOB‐513 wild type (Table S6). The main proteins upregulated in this mutant were associated with lipid transport and metabolism (I), and signal transduction mechanisms (T). As expected, among the down‐regulated proteins, we observed intracellular trafficking, secretion, and vesicular transport (U), cell wall/membrane/envelope biogenesis (M), and translation, ribosomal structure and biogenesis proteins (J) (Table S6 and Figure S2A,B).

The regulator AlgR was significantly decreased in MOB‐513‐*algR*::Tn relative to MOB‐513 wild type (Table S7 and Figure S3A,B). Proteins upregulated in this mutant were predominantly associated with inorganic ion transport and metabolism (P), and intracellular trafficking, secretion, and vesicular transport (U). Among the downregulated proteins, we observed defence mechanisms (V); energy production and conversion (C); and secondary metabolites biosynthesis, transport and catabolism proteins (Table S7 and Figure S3A,B).

The putative histidine kinase HK05390 was decreased in MOB‐513‐*hk05390*::Tn relative to MOB‐513 wild type (Table S8 and Figure S4A,B). The upregulated proteins in this mutant were associated with lipid transport and metabolism (I); transcription (K); coenzyme transport and metabolism (H); and signal transduction mechanisms (T). Among the proteins significantly decreased, we observed extracellular structures (W); defence mechanisms proteins (V); cell wall/membrane/envelope biogenesis (M); cell motility (N); energy production and conversion (C); and cell cycle control, cell division, chromosome partitioning proteins (D) (Table S8 and Figure S3A,B).

In MOB‐513‐*dgc05792p*::Tn, the abundance of the diguanylate cyclase Dgc05792 was significantly increased compared to the wild‐type strain (Table 2 and Table S9). In this mutant, the following functional categories were induced: signal transduction mechanisms (T); inorganic ion transport and metabolism (P); cell wall/membrane/envelope biogenesis (M); lipid transport and metabolism (I); amino acid transport and metabolism (E); and energy production and conversion (C). Furthermore, downregulated pathways included proteins involved in extracellular structures (W) and cell motility (N) (Table S9 and Figure S5A,B).

**TABLE 2:** Comparative proteomic analysis of MOB‐513 mutant strains.

Locus	Protein	Mean diff	*Q*‐value	COG^a^
*Proteins uniquely upregulated in MOB‐513‐dgc05792p::Tn*				
*PRES513_05792* (*dgc05792*)	Diguanylate cyclase, Dgc05792	2.97	4.56E‐04	T
*PRES513_04787/PRES513_04788* (*mop04787/mop04788*)	Manganese peroxidase, Mop04787/Mop04788	4.36	3.43E‐04	Q
*PRES513_03244* (*mco03244*)	Multicopper oxidase, Mco03244	3.73	0	DPM
*PRES513_02544* (*mco02544*)	Multicopper oxidase, Mco02544	4.97	0	DPM
*PRES513_05637*	Putative cytochrome c, CytC	2.68	7.73E‐04	EC
*PRES513_00629*	SSU ribosomal protein S14p (S29e), zinc‐independent	3.78	0.018214	J
*PRES513_01518*	Urea carboxylase‐related aminomethyltransferase	2.72	0	S
*PRES513_01406*	Hypothetical protein (YkvA family of proteins)	3.31	0.018026	S
*PRES513_05618*	Glycoprotein gp2	4.90	0	NA
*PRES513_05617*	Glycoprotein gp2	4.36	0	NA
*Proteins uniquely downregulated in MOB‐513‐pilC::Tn, MOB‐513‐algR::Tn and MOB‐513‐hk05390::Tn*				
*PRES513_04869*	Polyphosphate kinase 2			C

*Note:* Proteins are grouped according to whether they were uniquely increased in MOB‐513‐*dgc05792p*::Tn, or uniquely decreased in the three non‐oxidising mutants. Mean Diff, mean difference in abundance (*p* value ≤ 0.05).

Abbreviation: COG, cluster of orthologous groups functional category.

^a^ For the COG one‐letter codes are: *Information storage and processing*: [J] Translation, ribosomal structure and biogenesis; [A] RNA processing and modification; [K] Transcription; [L] Replication, recombination and repair; [B] Chromatin structure and dynamics. *Cellular processes and signalling*: [D] Cell cycle control, cell division, chromosome partitioning; [Y] Nuclear structure; [V] Defence mechanisms; [T] Signal transduction mechanisms; [M] Cell wall/membrane/envelope biogenesis; [N] Cell motility; [Z] Cytoskeleton; [W] Extracellular structures; [U] Intracellular trafficking, secretion, and vesicular transport; [O] Posttranslational modification, protein turnover, chaperones. *Metabolism*: [C] Energy production and conversion; [G] Carbohydrate transport and metabolism; [E] Amino acid transport and metabolism; [F] Nucleotide transport and metabolism; [H] Coenzyme transport and metabolism; [I] Lipid transport and metabolism; [P] Inorganic ion transport and metabolism; [Q] Secondary metabolites biosynthesis, transport and catabolism. Poorly characterised: [R] General function prediction only; [S] Function unknown; [NA] non assigned.

Interestingly, despite the distinct phenotype of the mutants, 10 proteins were significantly increased across all mutant strains, suggesting a common transposon‐associated effect (Table S10). These include: PRES513_04945 and PRES513_04488 involved in nitrate transport; PRES513_01521, PRES513_05745, PRES513_05746 and PRES513_05748, involved in urea transport and metabolism; PRES513_00823 involved in ribosome maturation and assembly; PRES513_02019 a 3‐phosphoglycerate kinase; and PRES513_04489 a response regulator. Moreover, one transcriptional regulator, PRES513_06150, was significantly decreased across all mutant strains.

We next compared the differential proteomic profiles of the hyper‐oxidising strain MOB‐513‐*dgc05792p*::Tn and the non‐oxidising mutants (*pilC*, *algR*, *hk05390*). Among the proteins uniquely upregulated in MOB‐513‐*dgc05792p*::Tn (Table 2), four Mn(II)‐oxidising proteins were identified. PRES513_04787 and PRES513_04788, previously identified as homologues of the Mn‐oxidising peroxidase MopA, and previously designated Mop7013 and Mop7014, respectively (Piazza et al. 2022). In this study, these proteins are referred to as Mop04787 and Mop04788, respectively, to reflect the updated genome annotation (Table S2). As reported previously (Piazza et al. 2022), these two genes are co‐transcribed from the same promoter region, and the corresponding proteins share a high degree of sequence similarity. Consequently, they cannot be reliably distinguished by proteomic analysis because the detected peptides have identical amino acid sequences in both proteins, making it impossible to determine whether the increased abundance corresponds to one or both proteins. Furthermore, PRES513_03244, homologue of the multicopper oxidase McoA from *putida* GB‐1, was renamed Mco03244; and PRES513_02544, homologous to CotA from *Bacillus pumilus* WH4 (Su et al. 2013), was renamed Mco02544. In addition, the abundance of PRES513_05637, a putative cytochrome c belonging to a protein class previously implicated in Mn(II) oxidation and extracellular electron transfer in MOB (de Vrind et al. 1998; Caspi et al. 1998) was also increased (Table 2). The increased production of these Mn(II)‐oxidising proteins likely explains the observed hyper‐oxidising phenotype.

Several other proteins were uniquely elevated in MOB‐513‐*dgc05792p*::Tn, suggesting additional contributions to Mn(II) oxidation. These include PRES513_00629 encoding SSU ribosomal protein S14p (S29e); PRES513_01518 encoding a Urea carboxylase‐related amino methyltransferase and PRES513_01406 encoding a hypothetical protein that belongs to the YkvA family of proteins, often associated with stress responses and redox‐related processes (Table 2). Finally, PRES513_05618 and PRES513_05617, encoding Glycoprotein gp2 proteins, which have a role in biofilm formation (Vozza et al. 2016), were significantly increased in MOB‐513‐*dgc05792p*::Tn and simultaneously decreased in *algR* and *pilC* mutants (Table 2).

Interestingly, the three non‐oxidising mutants showed a marked downregulation of PRES513_04869, which encodes a polyphosphate kinase 2 (PPK2), a pattern not observed in the hyper‐oxidising strain (Table 2). PPK2 enzymes use polyphosphate (polyP) as a phosphate donor to generate nucleotide triphosphates (NTPs) (Neville et al. 2022), and reduced PPK2 levels could therefore limit GTP availability for c‐di‐GMP synthesis. This is consistent with the lower intracellular c‐di‐GMP levels observed in these mutants.

The proteomic data also revealed reduced abundance of multiple Tfp proteins in the *pilC* mutant, beyond PilC itself, suggesting that loss of PilC has a broader detrimental impact on the expression and/or stability of other pili components (Table S6). Likewise, several Tfp proteins were downregulated in the *algR* and *hk05390* mutants (Tables S7 and S8). These results are consistent with the twitching motility phenotypes and TEM observations of these transposon mutants (Figures 2A and 3). Accordingly, the loss of Tfp formation in *pilC* and *algR* mutants, and the reduced Tfp production in the *hk05390* mutant, likely contribute to the biofilm defects observed in all three strains. A summary of the phenotypes associated with the transposon mutants characterised in this study is provided in Table 3.

**TABLE 3:** Summary of the characterised phenotypes of transposon insertion mutants affecting Mn(II) oxidation in MOB‐513.

Strain	Mn(II) oxidation	Biofilm formation	Twitching motility	Swimming motility	c‐di‐GMP levels	Effect of elevated c‐di‐GMP levels on Mn(II)‐oxidation^a^	Effect on Mn(II)‐oxidising proteins
WT	+	+	+	+	++	+++	NA
*pilC*::Tn	—	—	—	++	+	—	=
*algR*::Tn	—	—	—	++	+	—	=
*hk05390*::Tn	—	—	—	++	+	+++	=
*dgc05792p*::Tn	+++	++	+	—	+++	NA	++

*Note:* Phenotypes are indicated as follows: +, present; ++, increased; +++, strongly increased; –, reduced or absent; =, no change relative to the wild‐type strain.

Abbreviation: NA, not applicable.

^a^ Mn(II) oxidation phenotypes were re‐assessed in Tn mutants after increasing intracellular c‐di‐GMP levels via p*dgcB*‐mediated overexpression of DgcB.

## 4 Discussion

Despite the recognised biological importance of bacterial Mn(II) oxidation, the regulatory pathways governing this process remain poorly understood. *P. resinovorans* MOB‐513, an environmental isolate from a Mn‐rich habitat, represents a valuable model to dissect these mechanisms. Deeper insight into its regulatory networks could allow the development of improved biological filtration strategies for Mn(II) removal. To this end, we performed a random transposon mutagenesis screen in the environmental strain MOB‐513 (Piazza et al. 2019, 2022) to identify genetic determinants that modulate Mn(II) oxidation, either by abolishing or enhancing this phenotype. This approach uncovered several key factors influencing Mn(II) oxidation and provided new insights into the regulatory circuits underlying this process.

We found that genes involved in Tfp biogenesis and assembly, as well as transmembrane transport, influence Mn(II) oxidation in MOB‐513. Transposon insertions inactivating or disrupting these genes resulted in non‐oxidising phenotypes. Moreover, mutations affecting genes associated with c‐di‐GMP signalling, two‐component systems, and transcriptional regulation were linked to both non‐oxidising and hyper‐oxidising phenotypes, highlighting a strong association between these signalling networks and Mn(II) oxidation.

The second messenger c‐di‐GMP controls multiple cellular pathways in *Pseudomonas* species (Hengge et al. 2016), including Mn(II) oxidation (Piazza et al. 2022). In this study, we found that transposon mutants driving the expression of the native diguanylate cyclase *dgc05792* exhibited significantly higher Mn(II)‐oxidising activity than the wild‐type strain. This observation is consistent with our previous work demonstrating the importance of c‐di‐GMP in regulating Mn(II) oxidation (Piazza et al. 2022), although the specific molecular components mediating this regulation had remained unclear. In that study, intracellular c‐di‐GMP levels in MOB‐513 were globally manipulated through overexpression of the heterologous diguanylate cyclase *dgcB* and phosphodiesterase *pdeA* from *B. bronchiseptica* (Piazza et al. 2022). Under those conditions, elevated c‐di‐GMP levels increased the abundance of Mop04787 and Mop04788.

Here we extend these findings by identifying Dgc05792 as a native diguanylate cyclase associated with the regulation of Mn(II) oxidation. The *dgc05792p*::Tn strain displays elevated intracellular c‐di‐GMP levels and exhibits a hyper‐oxidising phenotype (Table 3). Consistent with this phenotype, activation of *dgc05792* increased the abundance of Mop04787 and Mop04788, as well as two multicopper oxidases Mco03244 and Mco02544. These observations suggest that the activation of Dgc05792 is associated with a specific c‐di‐GMP signalling pathway that differs from the effects observed following global manipulation of intracellular c‐di‐GMP levels. In addition, our proteomic analysis revealed increased abundance of PRES513_05637, a putative cytochrome c, in this hyper‐oxidising strain. Previous studies in *P. putida* GB‐1 showed that transposon insertions affecting cytochrome c biogenesis genes (*ccmF*, *ccmA* and *ccmE*) completely abolished Mn(II) oxidation, highlighting the essential role of c‐type cytochromes in this process (de Vrind et al. 1998; Caspi et al. 1998). In agreement, proteomics shows that this strain exhibited increased abundance of proteins associated with central metabolism and energy generation, whereas non‐oxidising mutants displayed signatures of metabolic imbalance. These findings support the notion that Mn(II) oxidation enhances cellular fitness, likely by providing an energetic advantage through the transfer of electrons from Mn(II) oxidation into the respiratory chain via c‐type cytochromes, as previously observed in another *Pseudomonas* sp. isolate (Ciancio Casalini et al. 2022).

The Mn‐peroxidases Mop04787 and Mop04788, the multicopper oxidases Mco03244 and Mco02544, and the cytochrome c PRES513_05637 were markedly more abundant in the hyper‐oxidising MOB‐513‐*dgc05792p*::Tn strain than in the wild‐type strain; however, no significant decrease in their abundance was observed in the non‐oxidising mutants. In our previous work, we showed that elevated c‐di‐GMP levels in MOB‐513 accelerate the onset of Mn(II) oxidation, suggesting that c‐di‐GMP regulates the timing and coordination of the oxidation machinery rather than simply increasing the abundance of Mn(II)‐oxidising proteins (Piazza et al. 2022). Given that the proteomic analysis was performed after 2 days of macrocolony growth, we speculate that the observed differences reflect a temporal shift in protein expression. Mn(II)‐oxidising proteins may be induced earlier in the hyper‐oxidising strain, whereas their expression is likely to remain at basal levels in the non‐oxidising mutants, as observed in the wild type (Piazza et al. 2022). Overall, these results suggest that Dgc05792 regulates a specific c‐di‐GMP signalling pathway associated with Mn(II) oxidation, expand the set of candidate Mn(II)‐oxidising enzymes under c‐di‐GMP control and suggest that MopA‐like proteins (Mop04787 and Mop04788), MCOs (Mco03244 and Mco02544), and cytochrome c (PRES513_05637) all contribute to the Mn(II) oxidation phenotype in MOB‐513.

Our results demonstrate that two‐component systems influence Mn(II) oxidation in MOB‐513. This is consistent with previous studies in the model strain *P. putida* GB‐1, in which the MnxS1/MnxS2/MnxR regulatory pathway was shown to be essential for Mn(II) oxidation (Geszvain and Tebo 2010). Our functional analysis predicted a large repertoire of TCSs in MOB‐513.

In our screen, three hyper‐oxidising mutants carried transposon insertions in genes predicted to encode components of distinct TCSs, suggesting that these systems negatively influence Mn(II) oxidation. Two of these mutants harboured insertions in *hk05573* (*PRES513_05573*), while a third carried an insertion in *hk05741* (*PRES513_05741*). Analysis for TCSs in MOB‐513 predicted that both genes encode hybrid histidine kinases, implying that the corresponding TCSs repress Mn(II) oxidation under specific conditions.

The predicted HK05390‐RR05389 and AlgZ‐AlgR TCS systems positively influence Mn(II) oxidation. Regarding the HK05390–RR05389 system, our results suggest that the response regulator RR05389 requires the histidine kinase HK05390 to exert its regulatory function, thereby revealing a novel mechanistic link between Mn(II) oxidation and c‐di‐GMP signalling. For the AlgZ‐AlgR system, similar to the established roles of AlgR in other *Pseudomonas* spp. (Tyanova et al. 2016), AlgR likely contributes to Mn(II) oxidation through its role in Tfp biogenesis. Consistent with this, the MOB‐513‐*algR*::Tn mutant behaved nearly identically to the MOB‐513‐*pilC*::Tn mutant. Both lacked detectable Tfp, displayed abolished twitching motility and enhanced swimming motility, and formed poor biofilms (Table 3). Importantly, neither Mn(II) oxidation nor biofilm formation could be restored in these mutants by elevating intracellular c‐di‐GMP through *dgcB* overexpression (Table 3), suggesting that Tfp functions downstream of c‐di‐GMP signalling in MOB‐513.

Interestingly, the TCSs associated with Mn(II) oxidation in MOB‐513 appear to be distinct from the MnxS1‐MnxS2‐MnxR system characterised in *P. putida* GB‐1 (Geszvain and Tebo 2010), suggesting that Mn(II) oxidation in MOB‐513 is regulated by a different two‐component signalling network. MnxS1 and MnxS2 have been reported to share 58% identity with each other but show little homology to characterised sensor kinase proteins and lack conserved domains such as PAS or HAMP. MnxR is predicted to function as a transcriptional regulator that interacts with *σ* ^54^‐containing RNA polymerase, similar to the *σ* ^54^‐dependent response regulator NtrC (Studholme and Dixon 2003). Furthermore, they lack any predicted diguanylate cyclase/phosphodiesterase domains (Geszvain and Tebo 2010). Collectively, these results highlight the diversity and complexity of TCS elements that shape the regulatory network controlling Mn(II) oxidation in *P. resinovorans* MOB‐513, likely integrating environmental cues through both c‐di‐GMP signalling and transcriptional regulation.

Our results revealed, for the first time, a strong dependence of Mn(II) oxidation on the integrity of the Tfp. The role of Tfp likely relates to its importance in initiating cell–cell contacts, promoting local increases in c‐di‐GMP, and enabling proper biofilm formation and architecture (Jin et al. 2011). Consistent with these functions, transposon mutants defective in Tfp biogenesis display reduced c‐di‐GMP levels and impaired biofilm formation, which together likely result in inefficient Mn(II) oxidation (Table 3). A correctly structured biofilm may be required to retain and concentrate extracellular Mn oxidases such as MCOs, MOPs, and c‐type cytochromes within the matrix, where Mn(II) oxidation occurs.

Beyond their well‐established roles in surface motility and biofilm development, Tfp have also been implicated in microbial interactions with manganese oxide, but exclusively in the context of Mn(III/IV) oxide acquisition or reduction, not oxidation. For example, in *Synechocystis* sp. PCC 6803, deletion of *pilA1* impaired growth on insoluble Mn oxides, suggesting that Tfp facilitate access to Mn bound in solid phases (Lamb and Hohmann‐Marriott 2017) and *Shewanella*, surface appendages, including Tfp‐like filaments and conductive nanowire, mediate extracellular electron transfer to insoluble Fe(III) and Mn oxides during reductive metabolism (Benedetti et al. 2016; Su et al. 2013). In this context, it is likely that Tfp contribute indirectly to Mn(II) redox physiology in *P. resinovorans* , for example by organising extracellular structures that channel electron flow from Mn(II) oxidation toward the respiratory chain, functioning in the opposite direction to their role during metal reduction in other systems.

The relevance of Tfp biogenesis in non‐oxidising mutants also suggests that Mn(II) oxidation may be coupled to surface‐associated behaviours, such as attachment to or interaction with the substrate. Consistent with this, a previous genetic screen for factors limiting Mn(II) oxidation in *P. putida* GB‐1 uncovered a link between Mn(II) oxidation and flagellar regulation and function (Geszvain et al. 2011). Using a similar random transposon mutagenesis strategy, it was found that hyper‐oxidising strains carried transposon insertions in genes encoding structural flagellar components (FliC, FliD, FlgE, and FlgL) as well as regulatory proteins (FlgM and FleN). This hyper‐oxidising phenotype was strongly dependent on growth conditions: solid media promoted earlier onset of Mn(II) oxidation, whereas liquid media had the opposite effect. These flagellar mutants were also defective in forming a pellicle biofilm at the air‐liquid interface. While disruption of flagellar function enhances Mn(II) oxidation in *P. putida* GB‐1, our findings indicate that the biogenesis of Tfp was important for efficient Mn(II) oxidation on solid media in MOB‐513. Together, these observations suggest that cellular structures involved in motility, surface sensing, and attachment influence the onset and efficiency of Mn(II) oxidation.

Our results reveal a strong association between bacterial Mn(II) oxidation and biofilm formation. Previous studies have shown that Mn(II) oxidation enhances bacterial tolerance to oxidative stress (Banh et al. 2013), while the accumulation of MnOx on the biofilm surface has been proposed to protect microbial communities from predation, viral attack (Geszvain et al. 2012), and ionising radiation (Daly et al. 2007). Biofilm architecture may also facilitate the activity and retention of Mn‐oxidising enzymes and the accumulation of MnOx. Because biofilms represent an important adaptive strategy that enhances bacterial persistence under adverse environmental conditions, Mn(II) oxidation may provide complementary fitness advantages to this bacterial lifestyle. Together, these traits may have evolved to improve bacterial survival by combining the structural and physiological benefits of the biofilm lifestyle with the protective functions associated with MnOx production. The reduced Mn(II)‐oxidising phenotype observed in mutants impaired in biofilm formation, together with the temporal separation of biofilm development and MnOx accumulation (Piazza et al. 2022), supports a model in which biofilm formation precedes and facilitates efficient Mn(II) oxidation in MOB‐513.

Our findings reveal an integrated regulatory network in which Tfp biogenesis, two‐component signalling and c‐di‐GMP homeostasis converge to control both Mn(II) oxidation and biofilm development in MOB‐513. This coordinated system underscores the tight coupling between surface structures, intracellular signalling, and extracellular redox processes required for efficient Mn(II) oxidation. Beyond advancing the fundamental understanding of manganese‐oxidising bacteria, these insights provide a foundation for designing new biotechnological strategies to enhance biological Mn(II) removal from groundwater.

## 5 Conclusions

This study identifies a regulatory network linking Type IV pili biogenesis, two‐component systems and c‐di‐GMP signalling to Mn(II) oxidation in *P. resinovorans* MOB‐513. We demonstrate that disruption of Tfp assembly or associated regulatory elements impairs biofilm formation and abolishes Mn(II) oxidation, while activation of the diguanylate cyclase Dgc05792 enhances c‐di‐GMP levels and promotes Mn(II)‐oxidising activity. Furthermore, we identify the HK05390‐RR05389 pair as a previously uncharacterised two‐component system that modulates c‐di‐GMP‐dependent phenotypes and Mn(II) oxidation. Together, these findings provide new mechanistic insight into the regulatory processes associated with bacterial Mn(II) oxidation and establish a framework for optimising biological manganese removal processes.

## Author Contributions

**Ainelén Piazza:** conceptualization, investigation, methodology, validation, visualization, writing – original draft, formal analysis. **Lucia Parra:** investigation, methodology, formal analysis. **Marilina Gaffuri:** investigation, methodology, formal analysis. **Jacob G. Malone:** funding acquisition, writing – review and editing, supervision, resources. **Jorgelina Ottado:** funding acquisition, writing – review and editing, supervision. **Natalia Gottig:** conceptualization, writing – original draft, writing – review and editing, funding acquisition, validation, supervision, methodology, visualization, resources.

## Funding

This work was supported by Agencia Nacional de Promoción Científica y Tecnológica (PICT2019‐01081), Consejo Nacional de Investigaciones Científicas y Técnicas (PIP‐1516), Agencia Santafesina de Ciencia, Tecnología e Innovación (DTT‐2023‐062), Fundación Williams, BBSRC Institute Strategic Programme (BB/X010996/1).

## Ethics Statement

This study did not involve human participants, vertebrate animals, or regulated clinical samples. All experimental procedures complied with institutional and national guidelines for laboratory biosafety.

## Conflicts of Interest

The authors declare no conflicts of interest.

## Supporting information


**Table S1:** Bacterial strains and plasmids used in this study.
**Table S2:** Correspondence between the previous and updated gene annotations.
**Table S3:** Oligonucleotides used in this study.
**Table S4:** Two‐component systems present in the MOB‐513 genome as predicted using P2RP software. Locus tags, protein class, family type, and predicted domain composition are shown. Protein classes include histidine kinases (HK), response regulators (RR), and phosphotransfer proteins (PP).
**Table S5:** Similarity between *P. resinovorans* MOB‐513 and *P. putida* GB‐1 TCSs. The table shows the best BLASTP matches for each response regulator and histidine kinase identified in the transposon screen. For each protein, the accession number of the closest homologue in GB‐1 is provided, together with percentage sequence identity (% identity) and E‐value.
**Table S6:** COG functional pathway analysis of proteins differentially abundant in the MOB‐513‐*pilC*::Tn mutant. Proteins are grouped according to whether they were increased or decreased relative to the wild‐type strain. Mean Diff, mean difference in abundance (*p* value ≤ 0.05); COG, Cluster of Orthologous Groups functional category.
**Table S7:** COG functional pathway analysis of proteins differentially abundant in the MOB‐513‐algR::Tn mutant. Proteins are grouped according to whether they were increased or decreased relative to the wild‐type strain. Mean Diff, mean difference in abundance (p value ≤ 0.05); COG, Cluster of Orthologous Groups functional category.
**Table S8:** COG functional pathway analysis of proteins differentially abundant in the MOB‐513‐*hk05390*::Tn mutant. Proteins are grouped according to whether they were increased or decreased relative to the wild‐type strain. Mean Diff, mean difference in abundance (*p* value ≤ 0.05); COG, Cluster of Orthologous Groups functional category.
**Table S9:** COG functional pathway analysis of proteins differentially abundant in the MOB‐513‐*dgc05792p*::Tn mutant. Proteins are grouped according to whether they were increased or decreased relative to the wild‐type strain. Mean Diff, mean difference in abundance (*p* value ≤ 0.05); COG, Cluster of Orthologous Groups functional category.
**Table S10:** Comparative proteomic analysis of MOB‐513 mutant strains. Proteins showing differential abundance across all transposon mutants are listed. Mean Diff, mean difference in protein abundance (*p* value ≤ 0.05); COG, Cluster of Orthologous Groups functional category.
**Figure S1:** Validation of the P_pel_A–msfGFP biosensor in MOB‐513 and c‐di‐GMP quantification in mutants overexpressing *dgcB*. (A) Performance of c‐di‐GMP biosensor in detecting both high and low cellular c‐di‐GMP levels in MOB‐513. msfGFP Fluorescence was measured in MOB‐513 strains co‐harbouring the biosensor plasmid and the p*dgcB* or p*pdeA* plasmids expressing the indicated proteins. The strain harbouring both the biosensor and pEmpty plasmids served as the control. Macrocolonies were on Lept+Mn agar at 28°C for 2 days. msfGFP Fluorescence (F) was recorded and normalised as described in material and methods Data represent mean ± standard deviation from triplicate measurements from at least three independent experiments. Statistical significance was assessed using one‐way ANOVA followed by Tukey's post hoc test. Asterisks denote statistically significant differences: * *p* < 0.0300. (B) Quantification of intracellular c‐di‐GMP levels in MOB‐513 Tn‐mutants transformed with p*dgcB* and in the WT strain transformed with p*dgc05792* or p*rr05389*. Macrocolonies were grown under the same conditions as indicated in (A), and msfGFP fluorescence was quantified identically. Data represent mean ± standard deviation from triplicate measurements from at least three independent experiments. Statistical significance was assessed using one‐way ANOVA followed by Tukey's post hoc test; no significant differences were detected relative to MOB‐513‐p*dgcB*.
**Figure S2:** (A) Volcano plot comparing protein abundance between MOB‐513 WT and MOB‐513‐*pilC*::Tn measured by label‐free proteomics (*n* = 4). A complete list of identified proteins and their predicted functions is provided in Table S6. PilC abundance is significantly reduced in MOB‐513‐*pilC*::Tn relative to WT. Differentially expressed proteins were defined as those showing a mean normalised abundance difference ≥ 2.5 or ≤ −2.5 between conditions (red vertical lines), with a *p* value ≤ 0.05 (dashed red horizontal line). (B) Proteins enriched in MOB‐513 WT (green bars) and MOB‐513‐*pilC*::Tn (red bars), grouped by COG assignments. Bar lengths indicate the number of proteins assigned to each category.
**Figure S3:** (A) Volcano plot comparing protein abundance between MOB‐513 WT and MOB‐513‐*algR*::Tn measured by label‐free proteomics (*n* = 4). A complete list of identified proteins and their predicted functions is provided in Table S7. AlgR abundance is significantly reduced in MOB‐513‐*algR*::Tn relative to WT. Differentially expressed proteins were defined as those showing a mean normalised abundance difference ≥ 2.5 or ≤ −2.5 between conditions (red vertical lines), with a *p* value ≤ 0.05 (dashed red horizontal line). (B) Proteins enriched in MOB‐513 WT (green bars) and MOB‐513‐*algR*::Tn (red bars), grouped by COG assignments. Bar lengths indicate the number of proteins assigned to each category.
**Figure S4:** (A) Volcano plot comparing protein abundance between MOB‐513 WT and MOB‐513‐*hk05390*::Tn measured by label‐free proteomics (*n* = 4). A complete list of identified proteins and their predicted functions is provided in Table S8. HK05390 abundance is significantly reduced in MOB‐513‐ *hk05390*::Tn relative to WT. Differentially expressed proteins were defined as those showing a mean normalised abundance difference ≥ 2.5 or ≤ −2.5 between conditions (red vertical lines), with a *p* value ≤ 0.05 (dashed red horizontal line). (B) Proteins enriched in MOB‐513 WT (green bars) and MOB‐513‐ *hk05390*::Tn (red bars), grouped by COG assignments. Bar lengths indicate the number of proteins assigned to each category.
**Figure S5:** (A) Volcano plot comparing protein abundance between MOB‐513 WT and MOB‐513‐*dgc05792* p::Tn measured by label‐free proteomics (*n* = 4). A complete list of identified proteins and their predicted functions is provided in Table S9. Dgc05792 abundance is significantly increased in MOB‐513‐ *dgc05792*p::Tn relative to WT and is associated with increased abundance multicopper oxidases Mco02544 and Mco03244; manganese peroxidases Mop04787 and Mop04788, and the cytochrome c PRES513_05637 (CytC). Differentially expressed proteins were defined as those showing a mean normalised abundance difference ≥ 2.5 or ≤ −2.5 between conditions (red vertical lines), with a *p* value ≤ 0.05 (dashed red horizontal line). (B) Proteins enriched in MOB‐513 WT (green bars) and MOB‐513‐ *dgc05792*p::Tn (red bars), grouped by COG assignments. Bar lengths indicate the number of proteins assigned to each category.

## Data Availability

The mass spectrometry proteomics data have been deposited to the ProteomeXchange Consortium via the PRIDE partner repository with the dataset identifier PXD075883 (10.6019/PXD075883). All other data supporting the findings of this study are available from the corresponding author upon request.

## References

[emi70423-bib-0001] Avila, D. S. , R. L. Puntel , and M. Aschner . 2013. “Manganese in Health and Disease.” Metal Ions in Life Sciences 13: 199–227. 10.1007/978-94-007-7500-8_7 PMC6589086 24470093

[emi70423-bib-0002] Banh, A. , V. Chavez , J. Doi , et al. 2013. “Manganese (Mn) Oxidation Increases Intracellular Mn in *Pseudomonas putida* GB‐1.” PLoS One 8, no. 10: e77835. 10.1371/journal.pone.0077835 PMC3798386 24147089

[emi70423-bib-0003] Barakat, M. , P. Ortet , and D. E. Whitworth . 2013. “P2RP: A Web‐Based Framework for the Identification and Analysis of Regulatory Proteins in Prokaryotic Genomes.” BMC Genomics 14: 269. 10.1186/1471-2164-14-269 PMC3637814 23601859

[emi70423-bib-0004] Baraquet, C. , K. Murakami , M. R. Parsek , and C. S. Harwood . 2012. “The FleQ Protein From *Pseudomonas aeruginosa* Functions as Both a Repressor and an Activator to Control Gene Expression From the Pel Operon Promoter in Response to c‐di‐GMP.” Nucleic Acids Research 40, no. 15: 7207–7218. 10.1093/nar/gks384 PMC3424551 22581773

[emi70423-bib-0005] Belete, B. , H. Lu , and D. J. Wozniak . 2008. “Pseudomonas Aeruginosa AlgR Regulates Type IV Pilus Biosynthesis by Activating Transcription of the fimU‐pilVWXY1Y2E Operon.” Journal of Bacteriology 190, no. 6: 2023–2030. 10.1128/JB.01623-07 PMC2258880 18178737

[emi70423-bib-0006] Benedetti, I. , V. de Lorenzo , and P. I. Nikel . 2016. “Genetic Programming of Catalytic *Pseudomonas putida* Biofilms for Boosting Biodegradation of Haloalkanes.” Metabolic Engineering 33: 109–118. 10.1016/j.ymben.2015.11.004 26620533

[emi70423-bib-0007] Bertani, G. 1951. “Studies on lysogenesis. I. The Mode of Phage Liberation by Lysogenic *Escherichia coli* .” Journal of Bacteriology 62, no. 3: 293–300. 10.1128/jb.62.3.293-300.1951 PMC386127 14888646

[emi70423-bib-0008] Boogerd, F. C. , and J. P. de Vrind . 1987. “Manganese Oxidation by *Leptothrix discophora* .” Journal of Bacteriology 169, no. 2: 489–494. 10.1128/jb.169.2.489-494.1987 PMC211803 3804969

[emi70423-bib-0009] Bradford, M. M. 1976. “A Rapid and Sensitive Method for the Quantitation of Microgram Quantities of Protein Utilizing the Principle of Protein–Dye Binding.” Analytical Biochemistry 72: 248–254. 10.1016/0003-2697(76)90527-3 942051

[emi70423-bib-0010] Caspi, R. , B. M. Tebo , and M. G. Haygood . 1998. “C‐Type Cytochromes and Manganese Oxidation in *Pseudomonas putida* MnB1.” Applied and Environmental Microbiology 64, no. 10: 3549–3555. 10.1128/aem.64.10.3549-3555.1998 PMC106463 9758766

[emi70423-bib-0011] Choi, K. H. , J. B. Gaynor , K. G. White , et al. 2005. “A Tn7‐Based Broad‐Range Bacterial Cloning and Expression System.” Nature Methods 2, no. 6: 443–448. 10.1038/nmeth765 15908923

[emi70423-bib-0012] Choi, K. H. , A. Kumar , and H. P. Schweizer . 2006. “A 10‐Min Method for Preparation of Highly Electrocompetent *Pseudomonas aeruginosa* Cells: Application for DNA Fragment Transfer Between Chromosomes and Plasmid Transformation.” Journal of Microbiological Methods 64, no. 3: 391–397. 10.1016/j.mimet.2005.06.001 15987659

[emi70423-bib-0013] Ciancio Casalini, L. , A. Piazza , F. Masotti , B. S. Garavaglia , J. Ottado , and N. Gottig . 2022. “Manganese Oxidation Counteracts the Deleterious Effect of Low Temperatures on Biofilm Formation in Pseudomonas sp. MOB‐449.” Frontiers in Molecular Biosciences 9: 1015582. 10.3389/fmolb.2022.1015582 PMC9634551 36339723

[emi70423-bib-0014] Daly, M. J. , E. K. Gaidamakova , V. Y. Matrosova , et al. 2007. “Protein Oxidation Implicated as the Primary Determinant of Bacterial Radioresistance.” PLoS Biology 5, no. 4: e92. 10.1371/journal.pbio.0050092 PMC1828145 17373858

[emi70423-bib-0015] de Vrind, J. P. , G. J. Brouwers , P. L. Corstjens , J. den Dulk , and E. W. de Vrind‐de Jong . 1998. “The Cytochrome c Maturation Operon Is Involved in Manganese Oxidation in *Pseudomonas putida* GB‐1.” Applied and Environmental Microbiology 64, no. 10: 3556–3562. 10.1128/aem.64.10.3556-3562.1998 PMC106464 9758767

[emi70423-bib-0016] Dombrecht, B. , J. Vanderleyden , and J. Michiels . 2001. “Stable RK2‐Derived Cloning Vectors for the Analysis of Gene Expression and Gene Function in Gram‐Negative Bacteria.” Molecular Plant‐Microbe Interactions 14, no. 3: 426–430. 10.1094/MPMI.2001.14.3.426 11277442

[emi70423-bib-0017] Geszvain, K. , C. Butterfield , R. E. Davis , et al. 2012. “The Molecular Biogeochemistry of Manganese(II) Oxidation.” Biochemical Society Transactions 40, no. 6: 1244–1248. 10.1042/BST20120229 23176462

[emi70423-bib-0018] Geszvain, K. , L. Smesrud , and B. M. Tebo . 2016. “Identification of a Third Mn(II) Oxidase Enzyme in *Pseudomonas putida* GB‐1.” Applied and Environmental Microbiology 82, no. 13: 3774–3782. 10.1128/AEM.00046-16 PMC4907182 27084014

[emi70423-bib-0019] Geszvain, K. , and B. M. Tebo . 2010. “Identification of a Two‐Component Regulatory Pathway Essential for Mn(II) Oxidation in *Pseudomonas putida* GB‐1.” Applied and Environmental Microbiology 76, no. 4: 1224–1231. 10.1128/AEM.02473-09 PMC2820985 20038702

[emi70423-bib-0020] Geszvain, K. , A. Yamaguchi , J. Maybee , and B. M. Tebo . 2011. “Mn(II) Oxidation in *Pseudomonas putida* GB‐1 Is Influenced by Flagella Synthesis and Surface Substrate.” Archives of Microbiology 193, no. 8: 605–614. 10.1007/s00203-011-0702-0 21479918

[emi70423-bib-0021] Guttenplan, S. B. , and D. B. Kearns . 2013. “Regulation of Flagellar Motility During Biofilm Formation.” FEMS Microbiology Reviews 37, no. 6: 849–871. 10.1111/1574-6976.12018 PMC3718880 23480406

[emi70423-bib-0022] Hengge, R. , A. Grundling , U. Jenal , R. Ryan , and F. Yildiz . 2016. “Bacterial Signal Transduction by Cyclic Di‐GMP and Other Nucleotide Second Messengers.” Journal of Bacteriology 198, no. 1: 15–26. 10.1128/JB.00331-15 PMC4686208 26055111

[emi70423-bib-0023] Jenal, U. , and J. Malone . 2006. “Mechanisms of Cyclic‐di‐GMP Signaling in Bacteria.” Annual Review of Genetics 40: 385–407. 10.1146/annurev.genet.40.110405.090423 16895465

[emi70423-bib-0024] Jin, F. , J. C. Conrad , M. L. Gibiansky , and G. C. Wong . 2011. “Bacteria Use Type‐IV Pili to Slingshot on Surfaces.” Proceedings of the National Academy of Sciences of the United States of America 108, no. 31: 12617–12622. 10.1073/pnas.1105073108 PMC3150923 21768344

[emi70423-bib-0025] Lamb, J. J. , and M. F. Hohmann‐Marriott . 2017. “Manganese Acquisition Is Facilitated by PilA in the Cyanobacterium Synechocystis sp. PCC 6803.” PLoS One 12, no. 10: e0184685. 10.1371/journal.pone.0184685 PMC5634550 29016622

[emi70423-bib-0026] Lindhout, T. , P. C. Y. Lau , D. Brewer , and J. S. Lam . 2009. “Truncation in the Core Oligosaccharide of Lipopolysaccharide Affects Flagella‐Mediated Motility in *Pseudomonas aeruginosa* PAO1 via Modulation of Cell Surface Attachment.” Microbiology (Reading) 155, no. Pt 10: 3449–3460. 10.1099/mic.0.030510-0 19589832

[emi70423-bib-0027] Liu, C. , R. Shi , M. S. Jensen , et al. 2024. “The Global1026 Regulation of c‐di‐GMP and cAMP in Bacteria.” mLife 3, no. 1: 42–56. 10.1002/mlf2.12104 PMC11139211 38827514

[emi70423-bib-0028] Malone, J. G. , T. Jaeger , C. Spangler , et al. 2010. “YfiBNR Mediates Cyclic Di‐GMP Dependent Small Colony Variant Formation and Persistence in *Pseudomonas aeruginosa* .” PLoS Pathogens 6, no. 3: e1000804. 10.1371/journal.ppat.1000804 PMC2837407 20300602

[emi70423-bib-0029] Martino, R. A. , D. C. Volke , A. H. Tenaglia , P. M. Tribelli , P. I. Nike1026l , and A. M. Smania . 2025. “Genetic Dissection of Cyclic Di‐GMP Signalling in *Pseudomonas aeruginosa* via Systematic Diguanylate Cyclase Disruption.” Microbial Biotechnology 18, no. 4: e70137. 10.1111/1751-7915.70137 PMC11963287 40172309

[emi70423-bib-0030] Monds, R. D. , P. D. Newell , J. C. Wagner , et al. 2010. “Di‐Adenosine Tetraphosphate (Ap4A) Metabolism Impacts Biofilm Formation by *Pseudomonas fluorescens* via Modulation of c‐di‐GMP‐Dependent Pathways.” Journal of Bacteriology 192, no. 12: 3011–3023. 10.1128/JB.01571-09 PMC2901679 20154123

[emi70423-bib-0031] Neville, N. , N. Roberge , and Z. Jia . 2022. “Polyphosphate Kinase 2 (PPK2) Enzymes: Structure, Function, and Roles in Bacterial Physiology and Virulence.” International Journal of Molecular Sciences 23, no. 2: 670. 10.3390/ijms23020670 PMC8776046 35054854

[emi70423-bib-0032] Okkotsu, Y. , A. S. Little , and M. J. Schurr . 2014. “The *Pseudomonas aeruginosa* AlgZR Two‐Component System Coordinates Multiple Phenotypes.” Frontiers in Cellular and Infection Microbiology 4: 82. 10.3389/fcimb.2014.00082 PMC4064291 24999454

[emi70423-bib-0033] O'Toole, G. A. , and R. Kolter . 1998. “Initiation of Biofilm Formation in *Pseudomonas fluorescens* WCS365 Proceeds via Multiple, Convergent Signalling Pathways: A Genetic Analysis.” Molecular Microbiology 28, no. 3: 449–461. 10.1046/j.1365-2958.1998.00797.x 9632250

[emi70423-bib-0034] Pacini, V. A. , A. Maria Ingallinella , and G. Sanguinetti . 2005. “Removal of Iron and Manganese Using Biological Roughing Up Flow Filtration Technology.” Water Research 39, no. 18: 4463–4475. 10.1016/j.watres.2005.08.027 16225901

[emi70423-bib-0035] Piazza, A. , L. Ciancio Casalini , V. A. Pacini , G. Sanguinetti , J. Ottado , and N. Gottig . 2019. “Environmental Bacteria Involved in Manganese(II) Oxidation and Removal From Groundwater.” Frontiers in Microbiology 10: 119. 10.3389/fmicb.2019.00119 PMC6396730 30853942

[emi70423-bib-0036] Piazza, A. , L. Parra , L. Ciancio Casalini , et al. 2022. “Cyclic Di‐GMP Signaling Links Biofilm Formation and Mn(II) Oxidation in *Pseudomonas resinovorans* .” MBio 13, no. 6: e0273422. 10.1128/mbio.02734-22 PMC9765421 36374078

[emi70423-bib-0037] Piazza, A. , C. M. A. Thompson , G. Chandra , et al. 2026. “A Novel Stress Response Pathway Mediates Biofilm Architecture in *Pseudomonas aeruginosa* .” PLoS Pathogens 22, no. 7: e1013832. 10.1371/journal.ppat.1013832 PMC13411936 42520047

[emi70423-bib-0038] Romling, U. , and M. Y. Galperin . 2015. “Bacterial Cellulose Biosynthesis: Diversity of Operons, Subunits, Products, and Functions.” Trends in Microbiology 23, no. 9: 545–557. 10.1016/j.tim.2015.05.005 PMC4676712 26077867

[emi70423-bib-0039] Sambrook, J. , and D. W. Russell . 2001. Molecular Cloning: A Laboratory Manual. 3rd ed. Cold Spring Harbour Laboratory.

[emi70423-bib-0040] Schindelin, J. , I. Arganda‐Carreras , E. Frise , et al. 2012. “Fiji: An Open‐Source Platform for Biological‐Image Analysis.” Nature Methods 9, no. 7: 676–682. 10.1038/nmeth.2019 PMC3855844 22743772

[emi70423-bib-0041] Schirmer, T. , and U. Jenal . 2009. “Structural and Mechanistic Determinants of c‐di‐GMP Signalling.” Nature Reviews. Microbiology 7, no. 10: 724–735. 10.1038/nrmicro2203 19756011

[emi70423-bib-0042] Semmler, A. B. , C. B. Whitchurch , and J. S. Mattick . 1999. “A Re‐Examination of Twitching Motility in *Pseudomonas aeruginosa* .” Microbiology (Reading) 145: 2863–2873. 10.1099/00221287-145-10-2863 10537208

[emi70423-bib-0043] Sisti, F. , D. G. Ha , G. A. O'Toole , D. Hozbor , and J. Fernandez . 2013. “Cyclic‐di‐GMP Signalling Regulates Motility and Biofilm Formation in *Bordetella bronchiseptica* .” Microbiology (Reading) 159: 869–879. 10.1099/mic.0.064345-0 PMC4085988 23475948

[emi70423-bib-0044] Sly, L. I. , M. C. Hodgkinson , and V. Arunpairojana . 1990. “Deposition of Manganese in a Drinking Water Distribution System.” Applied and Environmental Microbiology 56, no. 3: 628–639. 10.1128/aem.56.3.628-639.1990 PMC183397 2317040

[emi70423-bib-0045] Stock, A. M. , V. L. Robinson , and P. N. Goudreau . 2000. “Two‐Component Signal Transduction.” Annual Review of Biochemistry 69: 183–215. 10.1146/annurev.biochem.69.1.183 10966457

[emi70423-bib-0046] Studholme, D. J. , and R. Dixon . 2003. “Domain Architectures of sigma54‐Dependent Transcriptional Activators.” Journal of Bacteriology 185, no. 6: 1757–1767. 10.1128/JB.185.6.1757-1767.2003 PMC150144 12618438

[emi70423-bib-0047] Su, J. , P. Bao , T. Bai , et al. 2013. “CotA, a Multicopper Oxidase From *Bacillus pumilus* WH4, Exhibits Manganese‐Oxidase Activity.” PLoS One 8, no. 4: e60573. 10.1371/journal.pone.0060573 PMC3618234 23577125

[emi70423-bib-0048] Takhar, H. K. , K. Kemp , M. Kim , P. L. Howell , and L. L. Burrows . 2013. “The Platform Protein Is Essential for Type IV Pilus Biogenesis.” Journal of Biological Chemistry 288, no. 14: 9721–9728. 10.1074/jbc.M113.453506 PMC3617274 23413032

[emi70423-bib-0049] Thompson, C. M. A. , J. P. J. Hall , G. Chandra , et al. 2023. “Plasmids Manipulate Bacterial Behaviour Through Translational Regulatory Crosstalk.” PLoS Biology 21, no. 2: e3001988. 10.1371/journal.pbio.3001988 PMC9928087 36787297

[emi70423-bib-0050] Tyanova, S. , T. Temu , P. Sinitcyn , et al. 2016. “The Perseus Computational Platform for Comprehensive Analysis of (Prote)omics Data.” Nature Methods 13, no. 9: 731–740. 10.1038/nmeth.3901 27348712

[emi70423-bib-0051] Vozza, N. F. , P. L. Abdian , D. M. Russo , et al. 2016. “A *Rhizobium leguminosarum* CHDL‐ (Cadherin‐Like‐) Lectin Participates in Assembly and Remodeling of the Biofilm Matrix.” Frontiers in Microbiology 7: 1608. 10.3389/fmicb.2016.01608 PMC5061743 27790205

[emi70423-bib-0052] Walker, J. M. 1994. “The Bicinchoninic Acid (BCA) Assay for Protein Quantitation.” Methods in Molecular Biology 32: 5–8. 10.1385/0-89603-268-X:5 7951748

[emi70423-bib-0053] Whelton, A. J. , A. M. Dietrich , G. A. Burlingame , M. Schechs , and S. E. Duncan . 2007. “Minerals in Drinking Water: Impacts on Taste and Importance to Consumer Health.” Water Science and Technology 55, no. 5: 283–291. 10.2166/wst.2007.190 17489421

[emi70423-bib-0054] Xia, Y. , K. Li , J. Li , T. Wang , L. Gu , and L. Xun . 2019. “T5 Exonuclease‐Dependent Assembly Offers a Low‐Cost Method for Efficient Cloning and Site‐Directed Mutagenesis.” Nucleic Acids Research 47, no. 3: e15. 10.1093/nar/gky1169 PMC6379645 30462336

