# Supplementary material for: Identification of Molecular Pathways Underlying Mn(II) Oxidation in Pseudomonas resinovorans
Source: Environ Microbiol. 2026 Oct 1;28(10):e70423. doi: 10.1111/1462-2920.70423 (PMC13631088; doi:10.1111/1462-2920.70423)
Supplement: Supplementary file 1 [file EMI-28-e70423-s001.docx]

# **APPENDIX S1**

**Supplementary Tables**

**Table S1. Bacterial strains and plasmids used in this study.**

| **Strain** | **Description** | **Reference** |
| --- | --- | --- |
| ***Pseudomonas resinovorans*** | | |
| MOB-513 | Manganese oxidiser, wild type, Cm^R^  Accession number: GCA_986265995 | (1) |
| MOB-513-*pilC*::Tn | MOB-513 with the ALMAR3 transposon inserted into *pilC*, Cm^R^ Tc^R^ | This work |
| MOB-513-*algR*::Tn | MOB-513 with the ALMAR3 transposon inserted into *algR*, Cm^R^ Tc^R^ | This work |
| MOB-513-*hk05390*::Tn | MOB-513 with the ALMAR3 transposon inserted into *hk05390*, Cm^R^ Tc^R^ | This work |
| MOB-513-*dgc05792p*::Tn | MOB-513 with the ALMAR3 transposon inserted into *dgc05792 promoter*, Cm^R^ Tc^R^ | This work |
| MOB-513-*algR*::Tnc | MOB-513-*algR*::Tn containing p*algR*, Cm^R^ Tc^R^ Gm^R^ | This work |
| MOB-513-*hk05390*::Tnc | MOB-513-*hk05390*::Tn containing p*hk05390*, Cm^R^ Tc^R^ Gm^R^ | This work |
| MOB-513-pEmpty | MOB-513 containing pEmpty, Cm^R^ Gm^R^ | This work |
| MOB-513-p*dgcB* | MOB-513 containing p*dgcB*, Cm^R^ Gm^R^ | (2) |
| MOB-513-p*pdeA* | MOB-513 containing p*pdeA*, Cm^R^ Gm^R^ | (2) |
| MOB-513-*pilC*::Tn-pEmpty | MOB-513-*pilC*::Tn containing pEmpty, Cm^R^ Tc^R^ Gm^R^ | This work |
| MOB-513-*algR*::Tn-pEmpty | MOB-513-*algR*::Tn containing pEmpty, Cm^R^ Tc^R^ Gm^R^ | This work |
| MOB-513-*hk05390*::Tn-pEmpty | MOB-513-*hk05390*::Tn containing pEmpty, Cm^R^ Tc^R^ Gm^R^ | This work |
| MOB-513-*dgc05792p*::Tn-pEmpty | MOB-513-*dgc05792p*::Tn containing pEmpty, Cm^R^ Tc^R^ Gm^R^ | This work |
| MOB-513-*pilC*::Tn- p*dgcB* | MOB-513-*pilC*::Tn containing p*dgcB*, Cm^R^ Tc^R^ Gm^R^ | This work |
| MOB-513-*algR*::Tn-p*dgcB* | MOB-513-*algR*::Tn containing p*dgcB*, Cm^R^ Tc^R^ Gm^R^ | This work |
| MOB-513-*hk05390*::Tn-p*dgcB* | MOB-513-*hk05390*::Tn containing p*dgcB*, Cm^R^ Tc^R^ Gm^R^ | This work |
| MOB-513-*dgc05792p*::Tn-p*dgcB* | MOB-513-*dgc05792p*::Tn containing p*dgcB*, Cm^R^ Tc^R^ Gm^R^ | This work |
| MOB-513-p*dgc05792* | MOB-513 containing p*dgc05792*, Cm^R^ Tc^R^ Gm^R^ | This work |
| MOB-513-p*rr05389* | MOB-513 containing p*rr05389*, Cm^R^ Tc^R^ Gm^R^ | This work |
| MOB-513-*hk05390*::Tn-p*rr05389* | MOB-513-*hk05390*::Tn containing p*rr05389*, Cm^R^ Tc^R^ Gm^R^ | This work |
| ***Escherichia coli*** | | |
| DH5α λ*pir* | Cloning host; F*⁻* λ*⁻ endA1 glnX44(AS) thiE1 recA1 relA1 spoT1 gyrA96(Nalᴿ) rfbC1 deoR nupG Φ80(lacZΔM15) Δ(argF-lac) U169 hsdR17(rₖ⁻ mₖ⁺), λpir* lysogen | (3) |
| pALMAR3 | Insertion vector for Tc^R^ Mariner transposon | (4) |
| pEmpty | pBBR1MCS-5 (with *lacZ* promoter), Gm^R^ | (5) |
| p*algR* | pBBR1MCS-5 with *algR* inserted downstream the *lacZ* promoter, Gm^R^ | This work |
| p*hk05390* | pBBR1MCS-5 with *hk05390* inserted downstream the *lacZ* promoter, Gm^R^ | This work |
| p*dgcB* | pBBR1MCS-5 derived plasmid with *dgcB* inserted downstream the *nptII* promoter, Gm^R^ | (6) |
| p*dgc05792* | pBBR1MCS-5 with *dgc05792* inserted downstream the *lacZ* promoter, Gm^R^ | This work |
| p*rr05389* | pBBR1MCS-5 with *rr05389* inserted downstream the *lacZ* promoter, Gm^R^ | This work |
| pTnS1 | *ori*(R6K), TnsABCD transposase functions, Amp^R^ | (7) |
| pBG·PelA | plasmid pBG (Zobel et al. 2015) carrying the *PpelA* promoter as a *PpelA*(BDC2)→*msfGFP* transcriptional fusion, Km^R^ Gm^R^ | (8) |
| pBG·PelA·Sp | derivative of plasmid pBG·PelA with a Sp resistance cassette, Km^R^ Sp^R^ | (9) |

^a^*Antibiotic markers*: *Cm*, chloramphenicol; Tc, tetracycline; *Amp*, ampicillin; *Gm*, gentamicin; *Km*, kanamycin; *Sp*, streptomycin.

**Table S2. Correspondence between the previous and updated gene annotations.**

| **Locus tag**  **(this work)** | **Protein name**  **(This work)** | **Previous**  **locus tag (2)** | **Previous**  **protein name (2)** |
| --- | --- | --- | --- |
| *PRES513_04787* | Mop04787 | *Pres513_7013* | Mop7013 |
| *PRES513_04788* | Mop04788 | *Pres513_7014* | Mop7014 |
| *PRES513_03244* | Mco03244 | *Pres513_3296* | Mco3296 |
| *PRES513_02544* | Mco02544 | *Pres513_2061* | Mco2061 |
| *PRES513_01015* | PilZ | *Pres513_6471* | PilZ |

**Table S3. Oligonucleotides used in this study.**

| **Primer name** | **Sequence** (5'→3') | **Use** |
| --- | --- | --- |
| Arb-PCR | CGCAAACCAACCCTTGGCAG | Sequencing and mapping of transposon insertion sites (10) |
| Arb1 | GGCCAGCGAGCTAACGAGAC |  |
| Arb1b | GGCCAGCGAGCTAACGAGACNNNNGATAT |  |
| Arb1b Almar3-seq | ACATATCCATCGCGTCCGCC |  |
| rr05389-F | TATCGAATTCCTGCAGCCCAATGGCGCACCTGCAGCTTAAGG | Forward primer used for amplification and cloning of *rr05389* to construct p*rr05389* |
| rr05389-R | CTAGAACTAGTGGATCCCCCTCAGGAGGCAGGGCTGGGGG | Reverse primer used for amplification and cloning of *rr05389* to construct p*rr05389* |
| hk05390-F | TATCGAATTCCTGCAGCCCAATGGACGATCGACAATTGCC | Forward primer used for amplification and cloning of *hk05390* to construct p*hk05390* |
| hk05390-R | CTAGAACTAGTGGATCCCCCTTAAGCTGCAGGTGCGCCAT | Reverse primer used for amplification and cloning of *hk05390* to construct p*hk05390* |
| algR-F | TATCGAATTCCTGCAGCCCAATGAATGTCCTGATCGTCGAC | Forward primer used for amplification and cloning of *algR* to construct p*algR* |
| algR-R | CTAGAACTAGTGGATCCCCCCTATAGGTTGTTCATCAGTT | Reverse primer used for amplification and cloning of *algR* to construct p*algR* |
| dgc05792-F | TATCGAATTCCTGCAGCCCAATGGTGCCCCGTCCAAGCCTG | Forward primer used for amplification and cloning of *dgc05792* to construct p*dgc05792* |
| dgc05792-R | CTAGAACTAGTGGATCCCCCCTACAAAAGTCCGCTGCGAAT | Reverse primer used for amplification and cloning of *dgc05792* to construct p*dgc05792* |

**Table S4. Two-component systems present in the MOB-513 genome as predicted using P2RP software.** Locus tags, protein class, family type, and predicted domain composition are shown. Protein classes include histidine kinases (HK), response regulators (RR), and phosphotransfer proteins (PP).

| **Locus^a^** | **Class** | **Type** | **P2RP description** |
| --- | --- | --- | --- |
| *PRES513_05223* | RR | PleD | RR, PleD family,1 GGDEF |
| *PRES513_05224* | RR | CheB | RR, CheB family, 1 CheB_methylest |
| *PRES513_05225* | HK | CheA | HK, CheA 1 Hpt,1 HATPase c,1 CheW,1 RR |
| *PRES513_04134* | RR | OmpR | RR, OmpR family, 1 RR,1 Trans_reg_C |
| *PRES513_04720* | PP | HisKa | Phosphotransfer protein, 1 HisKA |
| *PRES513_04135* | HK | Classic | HK, Classic, 1 HisKA,1 HATPase_c |
| *PRES513_05001* | RR | NarL | RR, NarL family,1 HTH_LUXR |
| *PRES513_05002* | HK | Hybrid | HK, Hybrid, 4 HAMP,1 HisKA,1 HATPase_c,3 RR |
| *PRES513_05003* | HK | Hybrid | HK, Hybrid, 1 RR,1 HisKA_3,1 HATPase_c |
| *PRES513_05054* | HK | Classic | HK, Classic, 1 HAMP,1 HisKA,1 HATPase_c |
| *PRES513_05055* | RR | OmpR | RR, OmpR family, 1 RR,1 Trans_reg_C |
| *PRES513_05056* | HK | Unorthodox | HK, Unorthodox, 1 HAMP,1 HisKA,1 HATPase_c,1 RR Hpt |
| *PRES513_03733* | RR | CheY | RR, CheY family, 1 RR |
| *PRES513_03610* | HK | Classic | HK, Classic, 1 HisKA,1 HATPase_c |
| *PRES513_04330* | RR | CheY | RR, CheY family, 1 RR |
| *PRES513_02774* | RR | OmpR | RR, OmpR family, 1 RR,1 Trans_reg_C |
| *PRES513_02783* | HK | Classic | HK, Classic, 1 HAMP,1 HisKA,1 HATPase_c |
| *PRES513_04330* | RR | CheY | RR, CheY family, 1 RR |
| *PRES513_03258* | RR | NarL | RR, NarL family, 1 RR,1 GerE |
| *PRES513_04067* | RR | NarL | RR, NarL family, 1 RR,1 HTH_LUXR |
| *PRES513_04066* | HK | Unorthodox | HK, Unorthodox, 1 PBPb,1 SBP_bac_3,1 PAS_4,1 HisKA,1 HATPase_c,1 RR,1 Hpt |
| *PRES513_02575* | HK | Hybrid | HK, Hybrid, 1 HisKA,1 HATPase_c,1 RR |
| *PRES513_04039* | RR | CheV | RR, CheV family, 1 CheW,1 RR |
| *PRES513_00155* | RR | RpfG | RR, RpfG family, 1 RR,1 HDOD |
| *PRES513_01595* | RR | unclassified | RR, unclassified, 1 REC |
| *PRES513_06069* | RR | NtrC | RR, NtrC family, 1 RR,1 AAA_2,1 HTH_8 |
| *PRES513_06068* | HK | Classic | HK, Classic, 1 HisKA,1 HATPase_c |
| *PRES513_01765* | HK | Unorthodox | HK, Unorthodox, 1 MASE1,1 CHASE,1 PAS,1 HisKA,1 HATPase_c,2 RR,1 Hpt |
| *PRES513_01764* | RR | PleD | RR, PleD family, 1 RR,1 GGDEF |
| *PRES513_04629* | HK | Classic | HK, Classic, 1 PAS_4,1 HisKA,1 HATPase_c |
| *PRES513_04630* | RR | CheY | RR, CheY family, 1 RR |
| *PRES513_04631* | RR | CheY | RR, CheY family, 1 RR |
| *PRES513_04632* | RR | PleD_VieA | RR, PleD_VieA family, 2 PAS,2 GAF,1 PAS_4,1 PAS_3,1 GGDEF,1 EAL,1 RR |
| *PRES513_04649* | HK | Classic | HK, Classic, 1 HisKA,1 HATPase_c |
| *PRES513_04650* | RR | NtrC | RR, NtrC family, 1 RR,1 AAA,1 HTH_8 |
| *PRES513_04667* | RR | OmpR | RR, OmpR family, 1 RR,1 Trans_reg_C |
| *PRES513_04669* | HK | Classic | HK, Classic, 1 HAMP,1 HisKA,1 HATPase_c |
| *PRES513_01546* | HK | Classic | HK, Classic, 1 2CSK_N,1 HAMP,1 HisKA,1 HATPase_c |
| *PRES513_01545* | RR | OmpR | RR, OmpR family, 1 RR,1 Trans_reg_C |
| *PRES513_05389*  (*rr05389*) | RR | PleD_VieA | RR, PleD_VieA family, 1 RR,1 PAS,1 GGDEF,1 EAL |
| *PRES513_05390*  (*hk05390*) | HK | Hybrid | HK, Hybrid, 1 CHASE,1 PAS_4,1 PAS,1 HisKA,1 HATPase_c,3 RR |
| *PRES513_00561* | HK | Classic | HK, Classic, 1 SBP_bac_3,1 PAS_4,1 PAS_3,1 HisKA_3,1 HATPase_c |
| *PRES513_00562* | RR | NarL | RR, NarL family, 1 RR,1 HTH_LUXR |
| *PRES513_00404* | RR | NarL | RR, NarL family, 1 RR,1 GerE |
| *PRES513_00405* | HK | Unorthodox | HK, Unorthodox, 1 PBPb,1 SBP_bac_3,1 PAS_4,1 HisKA,1 HATPase_c,1 RR,1 Hpt |
| *PRES513_00406* | RR | VieA | RR, VieA family, 1 RR,1 EAL |
| *PRES513_00407* | RR | NarL | RR, NarL family, 1 RR,1 GerE |
| *PRES513_00446* | RR | OmpR | RR, OmpR family, 1 RR,1 Trans_reg_C |
| *PRES513_00447* | HK | Classic | HK, Classic, 1 HAMP,1 HisKA,1 HATPase_c |
| *PRES513_00455* | RR | CheY | RR, CheY family, 1 RR |
| *PRES513_00831* | HK | Hybrid | HK, Hybrid, 1 HisKA,1 HATPase_c,1 RR |
| *PRES513_04174* | RR | OmpR | RR, OmpR family, 1 RR,1 Trans_reg_C |
| *PRES513_04175* | HK | Classic | Probable incomplete HK , 1 HAMP,1 HATPase_c |
| *PRES513_05616* | HK | Classic | HK, Classic, 1 HisKA,1 HATPase_c |
| *PRES513_05615* | RR | NtrC | RR, NtrC family, 1 RR,1 AAA_5,1 HTH_8 |
| *PRES513_05573*  (*hk05573*) | HK | Hybrid | HK, Hybrid, 1 Cache_1,1 HAMP,1 PAS_3,1 HisKA,1 HATPase_c,1 RR |
| *PRES513_06244* | RR | CheY | RR, CheY family, 1 RR |
| *PRES513_06241* | HK | Classic | HK, Classic, 1 PAS,1 HisKA,1 HATPase_c |
| *PRES513_06240* | RR | OmpR | RR, OmpR family, 1 RR,1 Trans_reg_C |
| *PRES513_05825* | RR | PleD_VieA | RR, PleD_VieA family, 1 RR,1 PAS,1 GGDEF,1 EAL |
| *PRES513_05646* | RR | CheY | RR, CheY family, 1 RR |
| *PRES513_05647* | RR | NtrC | RR, NtrC family, 1 RR,1 AAA_5,1 HTH_8 |
| *PRES513_05648* | HK | Classic | HK, Classic, 1 HisKA,1 HATPase_c |
| *PRES513_05729* | RR | PleD | RR, PleD family, 1 RR,1 GGDEF |
| *PRES513_05741*  (*hk05741*) | HK | Hybrid | HK, Hybrid, 1 HisKA,1 HATPase_c,2 RR |
| *PRES513_00407* | RR | CheY | RR, CheY family, 1 RR |
| *PRES513_04779* | RR | CheV | RR, CheV family, 1 CheW,1 RR |
| *PRES513_04772* | RR | OmpR | RR, OmpR family, 1 RR,1 Trans_reg_C |
| *PRES513_04770* | HK | Unorthodox | HK, Unorthodox, 1 HAMP,1 HisKA,1 HATPase_c,1 RR,1 Hpt |
| *PRES513_04741* | HK | Classic | HK, Classic, 1 HisKA,1 HATPase_c |
| *PRES513_04744* | RR | NtrC | RR, NtrC family, 1 RR,1 AAA_5,1 HTH_8 |
| *PRES513_04741* | RR | NarL | RR, NarL family, 1 RR,1 HTH_LUXR |
| *PRES513_03713* | HK | Classic | HK, Classic, 1 Cache_2,1 HisKA_3,1 HATPase_c |
| *PRES513_03732* | HK | Classic | HK, Classic, 1 HisKA,1 HATPase_c |
| *PRES513_03733* | RR | NarL | RR, NarL family, 1 RR,1 GerE |
| *PRES513_05328* | HK | Classic | HK, Classic, 1 HAMP,1 HisKA,1 HATPase_c |
| *PRES513_05329* | RR | OmpR | RR, OmpR family, 1 RR,1 Trans_reg_C |
| *PRES513_01726* | RR | OmpR | RR, OmpR family, 1 RR,1 Trans_reg_C |
| *PRES513_01725* | HK | Classic | HK, Classic, 1 HAMP,1 HisKA,1 HATPase_c |
| *PRES513_01692* | RR | OmpR | RR, OmpR family, 1 RR,1 Trans_reg_C |
| *PRES513_01691* | HK | Classic | HK, Classic, 1 HAMP,1 HisKA,1 HATPase_c |
| *PRES513_04944* | RR | AmiR_NasR | RR, AmiR_NasR family, 1 RR,1 ANTAR |
| *PRES513_06159 (algZ)* | PP | HisKa | Phosphotransfer protein, 1 His_kinase |
| *PRES513_06158* (*algR*) | RR | LytTR | RR, LytTR family, 1 RR,1 LytTR |
| *PRES513_02101* | RR | OmpR | RR, OmpR family, 1 RR,1 Trans_reg_C |
| *PRES513_02102* | HK | Classic | HK, Classic, 1 HAMP,1 HisKA,1 HATPase_c |
| *PRES513_02105* | RR | OmpR | RR, OmpR family, 1 RR,1 Trans_reg_C |
| *PRES513_02106* | HK | Classic | HK, Classic, 1 HAMP,1 HisKA,1 HATPase_c |
| *PRES513_04336* | RR | OmpR | RR, OmpR family, 1 RR,1 Trans_reg_C |
| *PRES513_04335* | HK | Classic | Probable incomplete HK, 1 HAMP,1 HATPase_c |
| *PRES513_04331* | HK | Classic | HK, Classic, 1 HAMP,1 HisKA,1 HATPase_c |
| *PRES513_04330* | RR | OmpR | RR, OmpR family, 1 RR,1 Trans_reg_C |
| *PRES513_04324* | RR | RsbU | RR, RsbU family, 1 RR,1 SpoIIE |
| *PRES513_04861* | HK | Classic | HK, Classic, 1 HAMP,1 HisKA,1 HATPase_c |
| *PRES513_04862* | RR | OmpR | RR, OmpR family, 1 RR,1 Trans_reg_C |
| *PRES513_02250* | RR | YesN | RR, YesN family, 1 RR,2 HTH_AraC |
| *PRES513_02259* | RR | unclassified | RR, unclassified, 1 RR |
| *PRES513_05976* | RR | NtrC | RR, NtrC family, 1 RR,1 AAA_5,1 HTH_8 |
| *PRES513_05975* | HK | Classic | HK, Classic, 1 PAS,1 HisKA,1 HATPase_c |
| *PRES513_06307* | HK | Classic | HK, Classic, 1 HisKA,1 HATPase_c |
| *PRES513_06308* | RR | NtrC | RR, NtrC family, 1 RR,1 AAA,1 HTH_8 |
| *PRES513_00090* | HK | Classic | HK, Classic, 1 HAMP,1 PAS_4,1 HisKA,1 HATPase_c |
| *PRES513_00091* | RR | NtrC | RR, NtrC family, 1 RR,1 AAA,1 HTH_8 |
| *PRES513_00337* | HK | CheA | HK, CheA , 6 Hpt,1 H-kinase_dim,1 HATPase_c,1 CheW,1 RR |
| *PRES513_00333* | RR | CheY | RR, CheY family, 1 RR |
| *PRES513_00332* | RR | CheY | RR, CheY family, 1 RR |
| *PRES513_03483* | RR | NarL | RR, NarL family, 1 RR,1 HTH_LUXR |
| *PRES513_03484* | HK | Classic | HK, Classic, 1 PAS_4,1 HisKA,1 HATPase_c |
| *PRES513_03497* | RR | OmpR | RR, OmpR family, 1 RR,1 Trans_reg_C |
| *PRES513_03498* | HK | Classic | Probable incomplete HK, 1 HAMP,1 HATPase_c |
| *PRES513_01459* | HK | Hybrid | HK, Hybrid, 1 HisKA,1 HATPase_c,1 RR |
| *PRES513_02335* | RR | CheY | RR, CheY family, 1 REC |
| *PRES513_05081* | RR | NarL | RR, NarL family, 1 RR,1 HTH_LUXR |
| *PRES513_02916* | RR | NarL | RR, NarL family, 1 RR,1 GerE |
| *PRES513_04252* | RR | OmpR | RR, OmpR family, 1 RR,1 Trans_reg_C |
| *PRES513_04251* | HK | Hybrid | HK, Hybrid, 2 PAS_4,1 PAS,1 HisKA,1 HATPase_c,1 RR |
| *PRES513_04246* | HK | Hybrid | HK, Hybrid, 1 HAMP,1 HisKA,1 HATPase_c,1 RR |
| *PRES513_05517* | RR | unclassified | RR, unclassified, 1 RR,1 AAA_5 |
| *PRES513_05516* | HK | Classic | HK, Classic, 1 HisKA,1 HATPase_c |
| *PRES513_00405* | RR | CheY | RR, CheY family, 1 RR |
| *PRES513_03286* | HK | Hybrid | HK, Hybrid, 1 PAS_3,1 HisKA,1 HATPase_c,1 RR |
| *PRES513_00920* | RR | CheC | RR, CheC family, 1 RR,1 CheC |
| *PRES513_03256* | HK | Hybrid | HK, Hybrid, 2 PAS_4,1 HisKA,1 HATPase_c,1 RR |
| *PRES513_01007* | RR | PrrA | RR, PrrA family, 1 RR,1 HTH_8 |
| *PRES513_01008* | HK | Classic | HK, Classic, 1 HisKA,1 HATPase_c |
| *PRES513_01682* | RR | FrzZ | RR, FrzZ family, 2 RR |
| *PRES513_01681* | HK | Classic | Probable incomplete HK, 2 CBS,1 HATPase_c |
| *PRES513_01391* | HK | Hybrid | HK, Hybrid, 1 PAS,1 HisKA,1 HATPase_c,1 RR |
| *PRES513_06099* | RR | OmpR | RR, OmpR family, 1 RR,1 Trans_reg_C |
| *PRES513_06098* | HK | Classic | HK, Classic, 1 HAMP,1 HisKA,1 HATPase_c |
| *PRES513_04489* | RR | AmiR_NasR | RR, AmiR_NasR family, 1 RR,1 ANTAR |
| *PRES513_01163* | RR | PleD | RR, PleD family, 1 RR,1 GGDEF |
| *PRES513_01161* | HK | Classic | HK, Classic, 1 HAMP,1 HisKA,1 HATPase_c |
| *PRES513_04714* | RR | CheB | RR, CheB family, 1 RR,1 CheB_methylest |
| *PRES513_04715* | HK | CheA | HK, CheA, 1 Hpt,1 H-kinase_dim,1 HATPase_c,1 CheW |
| *PRES513_04717* | RR | CheY | RR, CheY family, 1 RR |
| *PRES513_04731* | PP | HPt | Phosphotransfer protein, 1 Hpt |
| *PRES513_04732* | RR | RsbU | RR, RsbU family, 1 RR,1 SpoIIE |
| *PRES513_01843* | RR | NarL | RR, NarL family, 1 RR,1 HTH_LUXR |
| *PRES513_01842* | HK | Classic | HK, Classic, 1 PAS_3,1 PAS,1 HisKA,1 HATPase_c |
| *PRES513_01075* | HK | Classic | HK, Classic, 1 HisKA,1 HATPase_c |
| *PRES513_01076* | RR | PrrA | RR, PrrA family, 1 RR,1 HTH_8 |
| *PRES513_00889* | HK | Hybrid | HK, Hybrid, 1 PAS_4,1 HisKA,1 HATPase_c,1 RR,1 PAS,1 GGDEF,1 EAL |

^a^ Genes interrupted by transposon insertions in our screen and their cognate partners are highlighted in red.

**Table S5. Similarity between *P. resinovorans* MOB-513 and *P. putida* GB-1 TCSs.** The table shows the best BLASTP matches for each response regulator and histidine kinase identified in the transposon screen. For each protein, the accession number of the closest homolog in GB-1 is provided, together with percentage sequence identity (% identity) and E-value.

| **MOB-513 regulator** | **Best hit in**  ***P. putida* GB-1** | **Locus in**  ***P. putida* GB-1** | **% Identity** | **E-value** |
| --- | --- | --- | --- | --- |
| HK05390 | Multi-sensor hybrid histidine kinase | PputGB1_2142 | 39.187% | 0.0 |
| RR05389 | Response regulator receiver modulated PAS/PAC sensor-containing diguanylate cyclase/ phosphodiesterase | PputGB1_5008 | 58.748 | 0.0 |
| AlgR | LytTR family two component transcriptional regulator | PputGB1_0210 | 79.839% | 0.0 |
| AlgZ | No significant similarity detected | — | — | — |
| HK05573 | Multi-sensor hybrid histidine kinase | PputGB1_2142 | 30.769 % | 1.22E-41 |
| HK05741 | Integral membrane sensor hybrid histidine kinase | PputGB1_4877 | 65.563 % | 0.0 |

**Table S6. COG functional pathway analysis of proteins differentially abundant in the MOB-513-*pilC*::Tn mutant.** Proteins are grouped according to whether they were increased or decreased relative to the wild-type strain. Mean Diff, mean difference in abundance (p-value ≤ 0.05); COG, Cluster of Orthologous Groups functional category.

| **Locus^a^** | **Protein** | **Mean Diff** | **Q-value** | **COG^b^** |
| --- | --- | --- | --- | --- |
| **Proteins significantly increased in the MOB-513-*pilC*::Tn mutant relative to MOB-513 wild type** | | | | |
| *PRES513_03489* | Acetyltransferase, GNAT family | 6.60 | 0 | EJOR |
| *PRES513_05887* | Universal stress protein UspA and related nucleotide-binding proteins | 5.69 | 0 | T |
| *PRES513_04945* | Nitrate ABC transporter, substrate-binding protein | 5.68 | 0 | P |
| *PRES513_05304* | CDP-abequose synthase (Fragment) | 5.17 | 0 | R |
| *PRES513_05748* | Urea ABC transporter, substrate binding protein UrtA | 5.09 | 0 | E |
| *PRES513_03007* | 3-oxoacyl-(acyl-carrier-protein) reductase, putative | 5.01 | 2.47E-04 | ICRM |
| *PRES513_04624* | Coproporphyrinogen III oxidase, oxygen-independent | 5.00 | 0 | H |
| *PRES513_06043* | UDP-glucose 6-dehydrogenase | 4.74 | 0 | MH |
| *PRES513_01521* | Urea carboxylase-related ABC transporter, periplasmic substrate-binding protein | 4.58 | 0 | P |
| *PRES513_02099* | Ribonucleotide reductase of class Ia (aerobic), beta subunit | 4.47 | 0 | F |
| *PRES513_01708* | Surface antigen | 4.44 | 0 | RGVS |
| *PRES513_06089* | Nitroreductase | 4.23 | 0 | C |
| *PRES513_00823* | Metal-dependent hydrolase YbeY, involved in rRNA and/or ribosome maturation and assembly | 4.21 | 2.33E-04 | J |
| *PRES513_04614* | Cytochrome c oxidase (cbb3-type) subunit CcoP | 4.20 | 0 | CP |
| *PRES513_05300* | hypothetical protein | 4.18 | 0 | T |
| *PRES513_00678* | Outer membrane protein W precursor | 4.07 | 1.80E-04 | M |
| *PRES513_03935* | Succinyl-CoA:3-ketoacid-coenzyme A transferase subunit B (EC 2.8.3.5) | 4.02 | 8.70E-04 | I |
| *PRES513_03936* | Succinyl-CoA:3-ketoacid-coenzyme A transferase subunit A | 4.00 | 0 | I |
| *PRES513_05746* | Urea carboxylase | 3.80 | 5.41E-04 | IERC |
| *PRES513_05745* | Allophanate hydrolase | 3.71 | 0 | J |
| *PRES513_01074* | probable periplasmic protein NMA1059 | 3.64 | 0.002051 | S |
| *PRES513_02019* | 3-phosphoglycerate kinase | 3.64 | 2.44E-04 | NA |
| *PRES513_06196* | D-amino acid dehydrogenase | 3.56 | 0 | E |
| *PRES513_01833* | (R)-3-hydroxydecanoyl-ACP:CoA transacylase PhaG (3-hydroxyacyl-CoA-acyl carrier protein transferase) | 3.37 | 0 | HRI |
| *PRES513_04488;*  *PRES513_04943* | Nitrate ABC transporter, substrate-binding protein | 3.32 | 0 | P |
| *PRES513_06262* | Alcohol dehydrogenase | 3.19 | 0 | GERC |
| *PRES513_05120* | hypothetical protein | 3.01 | 0.003845 | NA |
| *PRES513_04489* | Response regulator NasT | 3.00 | 3.32E-04 | TK |
| *PRES513_00773* | Ethanolamine ammonia-lyase light chain | 2.99 | 0 | E |
| *PRES513_05782* | Azurin | 2.87 | 0 | C |
| *PRES513_05656* | Protein PhnA | 2.86 | 0.016819 | R |
| *PRES513_05017* | Probable transcriptional regulatory protein YebC | 2.85 | 6.63E-04 | KJ |
| *PRES513_01953* | Phenylalanine-4-hydroxylase | 2.75 | 0 | E |
| *PRES513_01637* | Ferric iron ABC transporter, iron-binding protein | 2.75 | 0 | GHE |
| *PRES513_00344* | Protein yceI precursor | 2.75 | 0 | R |
| *PRES513_02152* | UPF0260 protein YcgN | 2.72 | 1.76E-04 | R |
| *PRES513_04948* | hypothetical protein | 2.71 | 7.35E-04 | NA |
| *PRES513_01638* | putative secreted oxidoreductase | 2.71 | 0 | E |
| *PRES513_05013* | Tol-Pal system-associated acyl-CoA thioesterase | 2.57 | 2.61E-04 | I |
| *PRES513_03939* | Intracellular protease | 2.53 | 0 | V |
| *PRES513_05376* | Type III effector HopPmaJ | 2.52 | 0.001527 | NA |
| **Proteins significantly decreased in the MOB-513-*pilC*::Tn mutant relative to MOB-513 wild type** | | | | |
| *PRES513_05898* | Type IV pilus biogenesis protein PilP | -2.52 | 0.024952 | NU |
| *PRES513_02280* | Tripartite tricarboxylate transporter TctC family | -2.53 | 0 | C |
| *PRES513_06219* | DNA-directed RNA polymerase omega subunit | -2.53 | 0.018173 | K |
| *PRES513_03481* | hypothetical protein | -2.60 | 0.0014 | NA |
| *PRES513_03905* | Major outer membrane lipoprotein I | -2.66 | 0.00277 | NA |
| *PRES513_03196* | hypothetical protein | -2.76 | 0.002505 | NA |
| *PRES513_03331* | Uncharacterized MFS-type transporter | -2.81 | 6.53E-04 | GEPR |
| *PRES513_04099* | Ribosome modulation factor | -2.82 | 0.003826 | J |
| *PRES513_04705* | hypothetical protein | -2.85 | 0 | NA |
| *PRES513_06203* | LSU ribosomal protein L33p, zinc-independent | -2.87 | 0.004427 | J |
| *PRES513_01211* | Type IV pilus biogenesis protein PilF | -2.87 | 1.78E-04 | MROCNWD |
| *PRES513_03019* | hypothetical protein | -2.89 | 0.001563 | NA |
| *PRES513_02282* | amidohydrolase 2 | -2.90 | 5.61E-04 | RG |
| *PRES513_03559* | Autotransporter adhesin | -2.96 | 0 | NA |
| *PRES513_03080* | Transcriptional regulator, AraC family | -2.97 | 2.40E-04 | K |
| *PRES513_05918* | Polyhydroxyalkanoate granule-associated protein PhaF | -2.99 | 0 | QT |
| *PRES513_02283* | 4-hydroxythreonine-4-phosphate dehydrogenase | -3.00 | 0.010579 | H |
| *PRES513_01641* | Membrane fusion component of tripartite multidrug efflux system | -3.05 | 0.001905 | VM |
| *PRES513_06096* | Ribosomal protein S6--L-glutamate ligase | -3.07 | 0 | EHJQMRI |
| *PRES513_04131* | putative lipoprotein | -3.07 | 6.12E-04 | NA |
| *PRES513_05212* | hypothetical protein | -3.20 | 0.004087 | NA |
| *PRES513_04393* | Transcriptional regulator, AraC family | -3.21 | 0.010795 | K |
| *PRES513_01886* | Putative outer membrane protein | -3.25 | 5.24E-04 | NA |
| *PRES513_01032*  (*pilC*) | Type IV fimbrial assembly protein PilC | -3.32 | 0.007988 | NU |
| *PRES513_02285* | L-lyxonate dehydratase | -3.33 | 5.21E-04 | MR |
| *PRES513_01991;*  *PRES513_05776* | Branched-chain amino acid transport system permease protein LivM | -3.41 | 0.002198 | EG |
| *PRES513_00069* | Methionine ABC transporter ATP-binding protein | -3.50 | 0.033284 | EMPDRQJVHCGO |
| *PRES513_05907* | LSU ribosomal protein L31p, zinc-dependent | -3.51 | 0.004325 | J |
| *PRES513_04417* | LSU ribosomal protein L20p | -3.80 | 0 | J |
| *PRES513_03844* | glutamine synthetase family protein | -3.88 | 0 | E |
| *PRES513_03750* | hypothetical protein | -3.93 | 0 | NA |
| *PRES513_04060* | Probable transmembrane protein | -4.03 | 0.001581 | J |
| *PRES513_03292* | hypothetical protein | -4.08 | 0.006794 | S |
| *PRES513_00258* | Cation/multidrug efflux pump | -4.21 | 2.68E-04 | NA |
| *PRES513_05618* | Glycoprotein gp2 | -4.42 | 3.38E-04 | NA |
| *PRES513_04869* | Polyphosphate kinase 2 | -4.47 | 0 | C |
| *PRES513_06198* | FAD dependent oxidoreductase | -5.05 | 0.011608 | E |
| *PRES513_03201* | hypothetical protein | -6.35 | 0 | C |
| *PRES513_06150* | Alginate regulatory protein AlgP, positive transcriptional regulator of AlgD | -6.87 | 0 | NA |
| *PRES513_01186* | Cytosol aminopeptidase PepA | -7.20 | 1.79E-04 | E |

^a^ Gene interrupted by transposon insertions in our screening is highlighted in red.

**^b^** For the COG one-letter codes are:

**INFORMATION STORAGE AND PROCESSING:** [J] Translation, ribosomal structure and biogenesis; [A] RNA processing and modification; [K] Transcription; [L] Replication, recombination and repair; [B] Chromatin structure and dynamics.

**CELLULAR PROCESSES AND SIGNALING:** [D] Cell cycle control, cell division, chromosome partitioning; [Y] Nuclear structure; [V] Defence mechanisms; [T] Signal transduction mechanisms; [M] Cell wall/membrane/envelope biogenesis; [N] Cell motility; [Z] Cytoskeleton; [W] Extracellular structures; [U] Intracellular trafficking, secretion, and vesicular transport; [O] Posttranslational modification, protein turnover, chaperones.

**METABOLISM:** [C] Energy production and conversion; [G] Carbohydrate transport and metabolism; [E] Amino acid transport and metabolism; [F] Nucleotide transport and metabolism; [H] Coenzyme transport and metabolism; [I] Lipid transport and metabolism; [P] Inorganic ion transport and metabolism; [Q] Secondary metabolites biosynthesis, transport and catabolism.

**POORLY CHARACTERIZED:** [R] General function prediction only; [S] Function unknown; [NA] non assigned.

**Table S7. COG functional pathway analysis of proteins differentially abundant in the MOB-513-*algR*::Tn mutant.** Proteins are grouped according to whether they were increased or decreased relative to the wild-type strain. Mean Diff, mean difference in abundance (p-value ≤ 0.05); COG, Cluster of Orthologous Groups functional category.

| **Locus^a^** | **Protein** | **Mean Diff** | **Q-value** | **COG^b^** |
| --- | --- | --- | --- | --- |
| **Proteins significantly increased in the MOB-513-*algR*::Tn mutant relative to MOB-513 wild type** | | | | |
| *PRES513_04945* | Nitrate ABC transporter, substrate-binding protein | 5.47 | 0.002842 | P |
| *PRES513_05748* | Urea ABC transporter, substrate binding protein UrtA | 4.77 | 0.0024 | E |
| *PRES513_01521* | Urea carboxylase-related ABC transporter, periplasmic substrate-binding protein | 4.62 | 0 | P |
| *PRES513_03571* | Uncharacterized protein ImpA | 4.20 | 0.001479 | U |
| *PRES513_05746* | Urea carboxylase | 4.00 | 0.00148 | IERC |
| *PRES513_00823* | Metal-dependent hydrolase YbeY, involved in rRNA and/or ribosome maturation and assembly | 3.75 | 8.85E-04 | J |
| *PRES513_03489* | Acetyltransferase, GNAT family | 3.67 | 0.002348 | EJOR |
| *PRES513_03731* | hypothetical protein | 3.53 | 0.001543 | NA |
| *PRES513_05745* | Allophanate hydrolase | 3.40 | 0 | J |
| *PRES513_04488;*  *PRES513_04943* | Nitrate ABC transporter, substrate-binding protein | 3.15 | 0.002074 | P |
| *PRES513_06175* | Ammonium transporter | 3.11 | 0.01036 | P |
| *PRES513_05304* | CDP-abequose synthase (Fragment) | 3.07 | 0 | R |
| *PRES513_04489* | Response regulator NasT | 3.05 | 8.37E-04 | TK |
| *PRES513_03775* | hypothetical protein | 2.30 | 0.013729 | R |
| *PRES513_01093* | Phosphocarrier protein, nitrogen regulation associated | 2.99 | 0.114628 | TG |
| *PRES513_02019* | 3-phosphoglycerate kinase | 2.93 | 0.018942 | NA |
| *PRES513_04432* | Putative regulatory protein | 2.92 | 0.022238 | RK |
| *PRES513_04614* | Cytochrome c oxidase (cbb3-type) subunit CcoP | 2.83 | 0.001263 | CP |
| *PRES513_05376* | Type III effector HopPmaJ | 2.73 | 0.00207 | NA |
| *PRES513_00634* | LSU ribosomal protein L30p (L7e) | 2.66 | 0.003195 | J |
| *PRES513_05300* | hypothetical protein | 2.61 | 0 | T |
| *PRES513_04624* | Coproporphyrinogen III oxidase, oxygen-independent | 2.55 | 0.00128 | H |
| *PRES513_05887* | Universal stress protein UspA and related nucleotide-binding proteins | 2.53 | 0.001509 | T |
| *PRES513_02878* | Cold shock protein of CSP family => CspD | 2.52 | 0.002316 | K |
| **Proteins significantly decreased in the MOB-513-*algR*::Tn mutant relative to MOB-513 wild type** | | | | |
| *PRES513_05617* | Glycoprotein gp2 | -2.58 | 0.00108 | NA |
| *PRES513_05772* | hypothetical protein | -2.64 | 7.66E-04 | C |
| *PRES513_02842* | Homocysteine S-methyltransferase | -2.70 | 0.001473 | E |
| *PRES513_04934* | Translation elongation factor G | -2.75 | 0.00225 | J |
| *PRES513_03625* | RND efflux system, inner membrane transporter | -2.80 | 0.017256 | VP |
| *PRES513_04936* | hypothetical protein | -2.84 | 0.002634 | HJRCIQ |
| *PRES513_06291* | Transcriptional regulator, AcrR family | -2.88 | 0.001317 | K |
| *PRES513_02252* | Predicted ATPase with chaperone activity, associated with Flp pilus assembly | -2.91 | 7.50E-04 | NA |
| *PRES513_03080* | Transcriptional regulator, AraC family | -2.94 | 9.91E-04 | K |
| *PRES513_00102* | Lysine-epsilon oxidase antimicrobial protein LodA | -3.06 | 0.003813 | NA |
| *PRES513_00297* | Cystathionine gamma-lyase | -3.08 | 0.002 | E |
| *PRES513_04869* | Polyphosphate kinase 2 | -3.22 | 0.002038 | C |
| *PRES513_04389* | Alkyl hydroperoxide reductase subunit C-like protein | -3.47 | 0.001289 | O |
| *PRES513_06150* | Alginate regulatory protein AlgP, positive transcriptional regulator of AlgD | -3.72 | 8.71E-04 | NA |
| *PRES513_02043* | hypothetical protein | -3.77 | 0.001584 | T |
| *PRES513_03750* | hypothetical protein | -3.78 | 0.001227 | NA |
| *PRES513_03078* | SAM-dependent methyltransferase | -3.81 | 0.002154 | HJCI |
| *PRES513_00296* | Cystathionine beta-synthase | -3.90 | 0 | ETK |
| *PRES513_05618* | Glycoprotein gp2 | -4.31 | 0.002769 | NA |
| *PRES513_00487* | hypothetical protein | -5.44 | 0.0018 | NA |
| *PRES513_06158*  (*algR*) | Alginate biosynthesis two-component system response regulator, AlgR | -5.55 | 0 | KT |
| *PRES513_03019* | hypothetical protein | -6.30 | 0.001137 | NA |
| *PRES513_04705* | hypothetical protein | -7.73 | 0 | NA |

^a^ Gene interrupted by transposon insertions in our screening is highlighted in red.

**^b^** For the COG one-letter codes are:

**INFORMATION STORAGE AND PROCESSING:** [J] Translation, ribosomal structure and biogenesis; [A] RNA processing and modification; [K] Transcription; [L] Replication, recombination and repair; [B] Chromatin structure and dynamics.

**CELLULAR PROCESSES AND SIGNALING:** [D] Cell cycle control, cell division, chromosome partitioning; [Y] Nuclear structure; [V] Defence mechanisms; [T] Signal transduction mechanisms; [M] Cell wall/membrane/envelope biogenesis; [N] Cell motility; [Z] Cytoskeleton; [W] Extracellular structures; [U] Intracellular trafficking, secretion, and vesicular transport; [O] Posttranslational modification, protein turnover, chaperones.

**METABOLISM:** [C] Energy production and conversion; [G] Carbohydrate transport and metabolism; [E] Amino acid transport and metabolism; [F] Nucleotide transport and metabolism; [H] Coenzyme transport and metabolism; [I] Lipid transport and metabolism; [P] Inorganic ion transport and metabolism; [Q] Secondary metabolites biosynthesis, transport and catabolism.

**POORLY CHARACTERIZED:** [R] General function prediction only; [S] Function unknown; [NA] non assigned.

**Table S8. COG functional pathway analysis of proteins differentially abundant in the MOB-513-*hk05390*::Tn mutant.** Proteins are grouped according to whether they were increased or decreased relative to the wild-type strain. Mean Diff, mean difference in abundance (p-value ≤ 0.05); COG, Cluster of Orthologous Groups functional category.

| **Locus^a^** | **Protein** | **Mean Diff** | **Q-value** | **COG^b^** |
| --- | --- | --- | --- | --- |
| **Proteins significantly increased in the MOB-513- *hk05390*::Tn mutant relative to MOB-513 wild type** | | | | |
| *PRES513_04945* | Nitrate ABC transporter, substrate-binding protein | 6.43 | 0 | P |
| *PRES513_01521* | Urea carboxylase-related ABC transporter, periplasmic substrate-binding protein | 5.77 | 0 | P |
| *PRES513_05748* | Urea ABC transporter, substrate binding protein UrtA | 4.96 | 0 | E |
| *PRES513_03775* | hypothetical protein | 4.73 | 4.65E-04 | R |
| *PRES513_02019* | 3-phosphoglycerate kinase | 4.16 | 0 | NA |
| *PRES513_00823;*  *PRES513_00823* | Metal-dependent hydrolase YbeY, involved in rRNA and/or ribosome maturation and assembly | 4.13 | 1.84E-04 | J |
| *PRES513_04488;*  *PRES513_04943* | Nitrate ABC transporter, substrate-binding protein | 4.03 | 0 | P |
| *PRES513_05746* | Urea carboxylase (EC 6.3.4.6) | 3.78 | 4.32E-04 | IERC |
| *PRES513_04489* | Response regulator NasT | 3.65 | 1.93E-04 | TK |
| *PRES513_05376* | Type III effector HopPmaJ | 3.58 | 0.001251 | NA |
| *PRES513_03571* | Uncharacterized protein ImpA | 3.42 | 0 | U |
| *PRES513_03612* | phytanoyl-CoA dioxygenase family protein | 3.29 | 1.39E-04 | Q |
| *PRES513_05084* | Putative NADPH-quinone reductase (modulator of drug activity B) | 3.26 | 0.013453 | NA |
| *PRES513_00156* | 2-hydroxychromene-2-carboxylate isomerase family protein | 3.22 | 1.37E-04 | QO |
| *PRES513_04773* | Transcriptional regulator, AcrR family | 3.02 | 0.002902 | K |
| *PRES513_06162* | DNA-binding protein inhibitor Id-2-related protein | 3.02 | 4.05E-04 | NA |
| *PRES513_05120* | hypothetical protein | 2.94 | 3.43E-04 | NA |
| *PRES513_01207* | Ferredoxin, 2Fe-2S | 2.88 | 1.80E-04 | C |
| *PRES513_05745* | Allophanate hydrolase | 2.78 | 0 | J |
| *PRES513_05329* | DNA-binding response regulator ColR | 2.74 | 1.77E-04 | TKD |
| *PRES513_00662* | Precorrin-6A reductase | 2.73 | 0.001919 | H |
| *PRES513_02226* | Transcriptional regulator, AcrR family | 2.67 | 0.003475 | K |
| *PRES513_02864* | Putative phosphatase | 2.67 | 1.87E-04 | R |
| *PRES513_04208* | Spermidine/putrescine import ABC transporter substrate-binding protein PotD | 2.65 | 1.32E-04 | EGH |
| *PRES513_01833* | (R)-3-hydroxydecanoyl-ACP:CoA transacylase PhaG | 2.63 | 0 | HRI |
| *PRES513_02139* | Glutathione peroxidase | 2.61 | 0.026046 | VIO |
| *PRES513_03616* | Putrescine ABC transporter putrescine-binding protein PotF | 2.57 | 0 | EGH |
| **Proteins significantly decreased in the MOB-513- *hk05390*::Tn mutant relative to MOB-513 wild type** | | | | |
| *PRES513_01885* | Putative outer membrane protein | -2.57 | 0 | NA |
| *PRES513_00123* | Glutaryl-CoA dehydrogenase | -2.58 | 0.001265 | NA |
| *PRES513_00995* | hypothetical protein | -2.83 | 0.006473 | NW |
| *PRES513_04393* | Transcriptional regulator, AraC family | -3.10 | 0.007118 | K |
| *PRES513_01421* | ABC transporter, ATP-binding protein | -3.12 | 0 | EGPMRHJVDCQO |
| *PRES513_01641* | Membrane fusion component of tripartite multidrug efflux system | -3.25 | 3.87E-04 | VM |
| *PRES513_04647* | Glutamate Aspartate transport ATP-binding protein GltL | -3.27 | 0.001069 | EMDPRJVHCQO |
| *PRES513_03844* | glutamine synthetase family protein | -3.29 | 5.15E-04 | E |
| *PRES513_03201* | hypothetical protein | -3.29 | 0.020629 | C |
| *PRES513_02285* | L-lyxonate dehydratase | -3.30 | 0 | MR |
| *PRES513_01886* | Putative outer membrane protein | -3.33 | 3.36E-04 | NA |
| *PRES513_04060* | Probable transmembrane protein | -3.41 | 5.28E-04 | J |
| *PRES513_00258* | Cation/multidrug efflux pump | -3.44 | 0.003277 | NA |
| *PRES513_05898* | Type IV pilus biogenesis protein PilP | -3.48 | 1.47E-04 | NU |
| *PRES513_04869* | Polyphosphate kinase 2 | -3.59 | 3.71E-04 | C |
| *PRES513_01186* | Cytosol aminopeptidase PepA | -3.61 | 0 | E |
| *PRES513_05390*  (*hk05390*) | Histidine Kinase, HK05390 | -4.00 | 4.89E-04 | TDK |
| *PRES513_06150* | Alginate regulatory protein AlgP, positive transcriptional regulator of AlgD | -4.06 | 0 | NA |

^a^ Gene interrupted by transposon insertions in our screening is highlighted in red.

**^b^** For the COG one-letter codes are:

**INFORMATION STORAGE AND PROCESSING:** [J] Translation, ribosomal structure and biogenesis; [A] RNA processing and modification; [K] Transcription; [L] Replication, recombination and repair; [B] Chromatin structure and dynamics.

**CELLULAR PROCESSES AND SIGNALING:** [D] Cell cycle control, cell division, chromosome partitioning; [Y] Nuclear structure; [V] Defence mechanisms; [T] Signal transduction mechanisms; [M] Cell wall/membrane/envelope biogenesis; [N] Cell motility; [Z] Cytoskeleton; [W] Extracellular structures; [U] Intracellular trafficking, secretion, and vesicular transport; [O] Posttranslational modification, protein turnover, chaperones.

**METABOLISM:** [C] Energy production and conversion; [G] Carbohydrate transport and metabolism; [E] Amino acid transport and metabolism; [F] Nucleotide transport and metabolism; [H] Coenzyme transport and metabolism; [I] Lipid transport and metabolism; [P] Inorganic ion transport and metabolism; [Q] Secondary metabolites biosynthesis, transport and catabolism.

**POORLY CHARACTERIZED:** [R] General function prediction only; [S] Function unknown; [NA] non assigned.

**Table S9. COG functional pathway analysis of proteins differentially abundant in the MOB-513-*dgc05792p*::Tn mutant.** Proteins are grouped according to whether they were increased or decreased relative to the wild-type strain. Mean Diff, mean difference in abundance (p-value ≤ 0.05); COG, Cluster of Orthologous Groups functional category.

| **Locus^a^** | **Protein** | **Mean Diff** | **Q-value** | **COG^b^** |
| --- | --- | --- | --- | --- |
| **Proteins significantly increased in the MOB-513-*dgc05792p*::Tn mutant** | | | | |
| *PRES513_04945* | Nitrate ABC transporter, substrate-binding protein | 5.79 | 0 | P |
| *PRES513_03489* | Acetyltransferase, GNAT family | 5.53 | 0 | EJOR |
| *PRES513_04614* | Cytochrome c oxidase (cbb3-type) subunit CcoP | 4.99 | 0 | CP |
| *PRES513_02544*  *(mco02544)* | Multicopper oxidase, Mco02544 | 4.97 | 0 | DPM |
| *PRES513_01521* | Urea carboxylase-related ABC transporter, periplasmic substrate-binding protein | 4.93 | 0 | P |
| *PRES513_05618* | Glycoprotein gp2 | 4.90 | 0 | NA |
| *PRES513_05748* | Urea ABC transporter, substrate binding protein UrtA | 4.70 | 0 | E |
| *PRES513_05304* | CDP-abequose synthase (Fragment) | 4.52 | 0 | R |
| *PRES513_05887* | Universal stress protein UspA and related nucleotide-binding proteins | 4.49 | 0 | T |
| *PRES513_02864* | Putative phosphatase | 4.40 | 0 | R |
| *PRES513_05746* | Urea carboxylase | 4.37 | 4.98E-04 | IERC |
| *PRES513_05617* | Glycoprotein gp2 | 4.36 | 0 | NA |
| *PRES513_04787/PRES513_04788 (mop04787/mop04788)* | Manganese peroxidase, Mop04787/Mop04788 | 4.36 | 3.43E-04 | Q |
| *PRES513_00823* | Metal-dependent hydrolase YbeY, involved in rRNA and/or ribosome maturation and assembly | 4.20 | 0 | J |
| *PRES513_00823* | Metal-dependent hydrolase YbeY, involved in rRNA and/or ribosome maturation and assembly | 4.20 | 0 | NA |
| *PRES513_04624* | Coproporphyrinogen III oxidase, oxygen-independent | 4.18 | 0 | H |
| *PRES513_05745* | Allophanate hydrolase | 3.93 | 0 | J |
| *PRES513_00629* | SSU ribosomal protein S14p (S29e), zinc-independent | 3.78 | 0.018214 | J |
| *PRES513_03244*  (*mco03244*) | Multicopper oxidase, Mco03244 | 3.73 | 0 | DPM |
| *PRES513_03007* | 3-oxoacyl-(acyl-carrier-protein) reductase, putative | 3.71 | 5.00E-04 | ICRM |
| *PRES513_03935* | Succinyl-CoA:3-ketoacid-coenzyme A transferase subunit B | 3.66 | 9.66E-04 | I |
| *PRES513_05300* | hypothetical protein | 3.64 | 0 | T |
| *PRES513_06089* | Nitroreductase | 3.59 | 0 | C |
| *PRES513_01708* | Surface antigen | 3.52 | 0 | RGVS |
| *PRES513_04488* | Nitrate ABC transporter, substrate-binding protein | 3.44 | 0 | P |
| *PRES513_02099* | Ribonucleotide reductase of class Ia (aerobic), beta subunit | 3.31 | 0 | F |
| *PRES513_01406* | hypothetical protein | 3.31 | 0.018026 | S |
| *PRES513_04489* | Response regulator NasT | 3.21 | 3.38E-04 | TK |
| *PRES513_06175* | Ammonium transporter | 3.16 | 0.008555 | P |
| *PRES513_01833* | (R)-3-hydroxydecanoyl-ACP:CoA transacylase PhaG | 3.11 | 0 | HRI |
| *PRES513_02019* | 3-phosphoglycerate kinase | 3.08 | 5.67E-04 | NA |
| *PRES513_05792*  *(dgc05792)* | Diguanylate cyclase, Dgc05792 | 2.97 | 4.56E-04 | T |
| *PRES513_00678* | Outer membrane protein W precursor | 2.74 | 7.40E-04 | M |
| *PRES513_05656* | Protein PhnA | 2.73 | 0.003828 | R |
| *PRES513_01518* | Urea carboxylase-related aminomethyltransferase | 2.72 | 0 | S |
| *PRES513_05637*  (*cytC*) | Putative cytochrome c, CytC | 2.68 | 7.73E-04 | EC |
| *PRES513_06262* | Alcohol dehydrogenase | 2.65 | 0 | GERC |
| *PRES513_03936* | Succinyl-CoA:3-ketoacid-coenzyme A transferase subunit A | 2.59 | 0 | I |
| *PRES513_05084* | Putative NADPH-quinone reductase | 2.53 | 0.065055 | NA |
| **Proteins significantly decreased in the MOB-513-*dgc05792p*::Tn mutant relative to MOB-513 wild type** | | | | |
| *PRES513_04419* | Translation initiation factor 3 | -2.50 | 0 | J |
| *PRES513_03080* | Transcriptional regulator, AraC family | -2.55 | 0.017984 | K |
| *PRES513_04099* | Ribosome modulation factor | -2.59 | 0.002219 | J |
| *PRES513_04393* | Transcriptional regulator, AraC family | -2.61 | 0.062204 | K |
| *PRES513_04647* | Glutamate Aspartate transport ATP-binding protein GltL | -2.67 | 0 | EMDPRJVHCQO |
| *PRES513_04060* | Probable transmembrane protein | -2.93 | 0.002315 | J |
| *PRES513_03193* | putative secreted protein | -2.93 | 0.099237 | QR |
| *PRES513_00995* | hypothetical protein | -2.93 | 0.01137 | NW |
| *PRES513_02282* | amidohydrolase 2 | -3.02 | 0 | RG |
| *PRES513_06293* | hypothetical protein | -3.12 | 5.12E-04 | J |
| *PRES513_02285* | L-lyxonate dehydratase | -3.41 | 4.66E-04 | MR |
| *PRES513_06150* | Alginate regulatory protein AlgP, positive transcriptional regulator of AlgD | -3.42 | 0 | NA |
| *PRES513_03750* | hypothetical protein | -3.61 | 5.83E-04 | NA |
| *PRES513_02562* | hypothetical protein | -4.12 | 0 | L |
| *PRES513_03201* | hypothetical protein | -5.58 | 0 | C |

^a^ Gene interrupted by transposon insertions in our screening and Mn-oxidising enzymes are highlighted in red.

**^b^**For the COG one-letter codes are:

**INFORMATION STORAGE AND PROCESSING:** [J] Translation, ribosomal structure and biogenesis; [A] RNA processing and modification; [K] Transcription; [L] Replication, recombination and repair; [B] Chromatin structure and dynamics.

**CELLULAR PROCESSES AND SIGNALING:** [D] Cell cycle control, cell division, chromosome partitioning; [Y] Nuclear structure; [V] Defence mechanisms; [T] Signal transduction mechanisms; [M] Cell wall/membrane/envelope biogenesis; [N] Cell motility; [Z] Cytoskeleton; [W] Extracellular structures; [U] Intracellular trafficking, secretion, and vesicular transport; [O] Posttranslational modification, protein turnover, chaperones.

**METABOLISM:** [C] Energy production and conversion; [G] Carbohydrate transport and metabolism; [E] Amino acid transport and metabolism; [F] Nucleotide transport and metabolism; [H] Coenzyme transport and metabolism; [I] Lipid transport and metabolism; [P] Inorganic ion transport and metabolism; [Q] Secondary metabolites biosynthesis, transport and catabolism.

**POORLY CHARACTERIZED:** [R] General function prediction only; [S] Function unknown; [NA] non assigned.

**Table S10. Comparative proteomic analysis of MOB-513 mutant strains.** Proteins showing differential abundance across all transposon mutants are listed. Mean Diff, mean difference in protein abundance (p-value ≤ 0.05); COG, Cluster of Orthologous Groups functional category.

| **Locus** | **Protein** | **Mean Diff** | **Q-value** | **COG*** |
| --- | --- | --- | --- | --- |
| **Shared differentially abundant proteins across all transposon mutants** | | | | |
| *PRES513_04945* | Nitrate ABC transporter, substrate-binding protein | 5.79 | 0 | P |
| *PRES513_04488* | Nitrate ABC transporter, substrate-binding protein | 3.44 | 0 | P |
| *PRES513_01521* | Urea carboxylase-related ABC transporter, periplasmic substrate-binding protein | 4.93 | 0 | P |
| *PRES513_05745* | Allophanate hydrolase | 3.93 | 0 | J |
| *PRES513_05746* | Urea carboxylase | 4.37 | 4.98E-04 | IERC |
| *PRES513_05748* | Urea ABC transporter, substrate binding protein UrtA | 4.70 | 0 | E |
| *PRES513_00823* | Metal-dependent hydrolase YbeY, involved in rRNA and/or ribosome maturation and assembly | 4.20 | 0 | J |
| *PRES513_02019* | 3-phosphoglycerate kinase | 3.08 | 5.67E-04 | NA |
| *PRES513_04489* | Response regulator NasT | 3.21 | 3.38E-04 | TK |
| *PRES513_06150* | Alginate regulatory protein AlgP, positive transcriptional regulator of AlgD | -3.42 | 0 | NA |

*****For the COG one-letter codes are:

**INFORMATION STORAGE AND PROCESSING:** [J] Translation, ribosomal structure and biogenesis; [A] RNA processing and modification; [K] Transcription; [L] Replication, recombination and repair; [B] Chromatin structure and dynamics.

**CELLULAR PROCESSES AND SIGNALING:** [D] Cell cycle control, cell division, chromosome partitioning; [Y] Nuclear structure; [V] Defence mechanisms; [T] Signal transduction mechanisms; [M] Cell wall/membrane/envelope biogenesis; [N] Cell motility; [Z] Cytoskeleton; [W] Extracellular structures; [U] Intracellular trafficking, secretion, and vesicular transport; [O] Posttranslational modification, protein turnover, chaperones.

**METABOLISM:** [C] Energy production and conversion; [G] Carbohydrate transport and metabolism; [E] Amino acid transport and metabolism; [F] Nucleotide transport and metabolism; [H] Coenzyme transport and metabolism; [I] Lipid transport and metabolism; [P] Inorganic ion transport and metabolism; [Q] Secondary metabolites biosynthesis, transport and catabolism.

**POORLY CHARACTERIZED:** [R] General function prediction only; [S] Function unknown; [NA] non assigned.

**Supplementary Figures**

**Figure S1**

**Figure S1. Validation of the P_pel_A–msfGFP biosensor in MOB-513 and c-di-GMP quantification in mutants overexpressing *dgcB***.

**(A)** Performance of c-di-GMP biosensor in detecting both high and low cellular c-di-GMP levels in MOB-513. msfGFP Fluorescence was measured in MOB-513 strains co-harbouring the biosensor plasmid and the p*dgcB* or p*pdeA* plasmids expressing the indicated proteins. The strain harbouring both the biosensor and pEmpty plasmids served as the control. Macrocolonies were on Lept+Mn agar at 28°C for 2 days. msfGFP Fluorescence (F) was recorded and normalized as described in material and methods Data represent mean ± standard deviation from triplicate measurements from at least three independent experiments. Statistical significance was assessed using one-way ANOVA followed by Tukey’s post hoc test. Asterisks denote statistically significant differences: * P < 0.0300.

**(B)** Quantification of intracellular c-di-GMP levels in MOB-513 Tn-mutants transformed with p*dgcB* and in the WT strain transformed with p*dgc05792* or p*rr05389*. Macrocolonies were grown under the same conditions as indicated in A, and msfGFP fluorescence was quantified identically. Data represent mean ± standard deviation from triplicate measurements from at least three independent experiments. Statistical significance was assessed using one-way ANOVA followed by Tukey’s post hoc test; no significant differences were detected relative to MOB-513-p*dgcB*.

**Figure S2**

**Figure S2. A.** Volcano plot comparing protein abundance between MOB-513 WT and MOB-513-*pilC*::Tn measured by label-free proteomics (n = 4). A complete list of identified proteins and their predicted functions is provided in Table S6. PilC abundance is significantly reduced in MOB-513-*pilC*::Tn relative to WT. Differentially expressed proteins were defined as those showing a mean normalised abundance difference ≥ 2.5 or ≤ −2.5 between conditions (red vertical lines), with a p-value ≤ 0.05 (dashed red horizontal line). **B.** Proteins enriched in MOB-513 WT (green bars) and MOB-513-*pilC*::Tn (red bars), grouped by COG assignments. Bar lengths indicate the number of proteins assigned to each category.

**Figure S3**

**Figure S3. A.** Volcano plot comparing protein abundance between MOB-513 WT and MOB-513-*algR*::Tn measured by label-free proteomics (n = 4). A complete list of identified proteins and their predicted functions is provided in Table S7. AlgR abundance is significantly reduced in MOB-513-*algR*::Tn relative to WT. Differentially expressed proteins were defined as those showing a mean normalised abundance difference ≥ 2.5 or ≤ −2.5 between conditions (red vertical lines), with a p-value ≤ 0.05 (dashed red horizontal line). **B.** Proteins enriched in MOB-513 WT (green bars) and MOB-513-*algR*::Tn (red bars), grouped by COG assignments. Bar lengths indicate the number of proteins assigned to each category.

**Figure S4**

**Figure S4. A.** Volcano plot comparing protein abundance between MOB-513 WT and MOB-513-*hk05390*::Tn measured by label-free proteomics (n = 4). A complete list of identified proteins and their predicted functions is provided in Table S8. HK05390 abundance is significantly reduced in MOB-513- *hk05390*::Tn relative to WT. Differentially expressed proteins were defined as those showing a mean normalised abundance difference ≥ 2.5 or ≤ −2.5 between conditions (red vertical lines), with a p-value ≤ 0.05 (dashed red horizontal line). **B.** Proteins enriched in MOB-513 WT (green bars) and MOB-513- *hk05390*::Tn (red bars), grouped by COG assignments. Bar lengths indicate the number of proteins assigned to each category.

**Figure S5**

**Figure S5. A.** Volcano plot comparing protein abundance between MOB-513 WT and MOB-513-*dgc05792* p::Tn measured by label-free proteomics (n = 4). A complete list of identified proteins and their predicted functions is provided in Table S9. Dgc05792 abundance is significantly increased in MOB-513- *dgc05792*p::Tn relative to WT and is associated with increased abundance multicopper oxidases Mco02544 and Mco03244; manganese peroxidases Mop04787 and Mop04788, and the cytochrome c PRES513_05637 (CytC). Differentially expressed proteins were defined as those showing a mean normalised abundance difference ≥ 2.5 or ≤ −2.5 between conditions (red vertical lines), with a p-value ≤ 0.05 (dashed red horizontal line). **B.** Proteins enriched in MOB-513 WT (green bars) and MOB-513- *dgc05792*p::Tn (red bars), grouped by COG assignments. Bar lengths indicate the number of proteins assigned to each category.

**References**

1.	Piazza A, Ciancio Casalini L, Pacini VA, Sanguinetti G, Ottado J, Gottig N. Environmental Bacteria Involved in Manganese(II) Oxidation and Removal From Groundwater. Front Microbiol. 2019;10:119.

2.	Piazza A, Parra L, Ciancio Casalini L, Sisti F, Fernandez J, Malone JG, et al. Cyclic di-GMP Signaling Links Biofilm Formation and Mn(II) Oxidation in Pseudomonas resinovorans. mBio. 2022;13(6):e0273422.

3.	Woodcock DM, Crowther PJ, Doherty J, Jefferson S, DeCruz E, Noyer-Weidner M, et al. Quantitative evaluation of Escherichia coli host strains for tolerance to cytosine methylation in plasmid and phage recombinants. Nucleic Acids Res. 1989;17(9):3469-78.

4.	Malone JG, Jaeger T, Spangler C, Ritz D, Spang A, Arrieumerlou C, et al. YfiBNR mediates cyclic di-GMP dependent small colony variant formation and persistence in Pseudomonas aeruginosa. PLoS Pathog. 2010;6(3):e1000804.

5.	Abramson J, Adler J, Dunger J, Evans R, Green T, Pritzel A, et al. Addendum: Accurate structure prediction of biomolecular interactions with AlphaFold 3. Nature. 2024;636(8042):E4.

6.	Sisti F, Ha DG, O'Toole GA, Hozbor D, Fernandez J. Cyclic-di-GMP signalling regulates motility and biofilm formation in Bordetella bronchiseptica. Microbiology (Reading). 2013;159(Pt 5):869-79.

7.	Choi KH, Gaynor JB, White KG, Lopez C, Bosio CM, Karkhoff-Schweizer RR, et al. A Tn7-based broad-range bacterial cloning and expression system. Nat Methods. 2005;2(6):443-8.

8.	Benedetti I, de Lorenzo V, Nikel PI. Genetic programming of catalytic Pseudomonas putida biofilms for boosting biodegradation of haloalkanes. Metab Eng. 2016;33:109-18.

9.	Martino RA, Volke DC, Tenaglia AH, Tribelli PM, Nikel PI, Smania AM. Genetic Dissection of Cyclic di-GMP Signalling in Pseudomonas aeruginosa via Systematic Diguanylate Cyclase Disruption. Microb Biotechnol. 2025;18(4):e70137.

10.	O'Toole GA, Kolter R. Initiation of biofilm formation in Pseudomonas fluorescens WCS365 proceeds via multiple, convergent signalling pathways: a genetic analysis. Mol Microbiol. 1998;28(3):449-61.
